# Bone marrow mesenchymal stem cells-derived exosomes ameliorate doxorubicin-induced nephrotoxicity via the modulation of pyroptosis-associated signaling

**DOI:** 10.1186/s10020-026-01655-6

**Published:** 2026-10-31

**Authors:** Reem K. Shahin, Ola Elazazy, Sara A. Wahdan, Sherihan G. AbdelHamid

**Affiliations:** 1 Biochemistry Department, Faculty of Pharmacy, Badr University in Cairo (BUC), Badr City, 11829 Cairo Egypt; 2 Pharmacology and Toxicology Department, Faculty of Pharmacy, Ain Shams University, Cairo, 11566 Egypt; 3 Biochemistry and Molecular Biology Department, Faculty of Pharmacy, Ain Shams University, Cairo, 11566 Egypt

**Keywords:** Doxorubicin, Nephrotoxicity, Exosomes, Pyroptosis, ELAVL1, miR-146a-5p

## Abstract

Doxorubicin (DOX) remains a cornerstone chemotherapeutic agent whose clinical application is constrained by nephrotoxicity, with no therapeutic interventions currently available. This study aimed to delineate the ameliorative potential of bone marrow mesenchymal stem cells (BM-MSCs) derived exosomes (EXO) in an experimental model of DOX-induced acute kidney injury (AKI). Twenty-four rats were divided into 4 groups: Control, DOX intoxicated, DOX + EXO, and EXO only groups. Nephrotoxicity was induced in the DOX and DOX + EXO groups using a single dose of DOX (15 mg/ kg *i.p.*). After 24 h, DOX + EXO and EXO only groups were given BM-MSCs-derived exosomes (100 μg *i.v.*). DOX was associated with a decline in renal function, activation of oxidative stress, inflammation, and modulation of pyroptosis-associated inflammasome-related signaling. BM-MSCs-derived exosomes significantly improved renal function, as evidenced by histopathological examination, reduction in KIM-1, blood urea nitrogen (BUN), serum creatinine, and renal somatic index (RSI). Exosomes mitigated oxidative stress by enhancing endogenous antioxidant catalase activity and reducing the lipid peroxidation marker MDA, while also suppressing inflammatory mediators, as shown by a reduction in IL-18 and IL-1β. Furthermore, exosomal treatment attenuated the expression of NLRP3, caspase-1 (CASP-1), and gasdermin-D (GSDMD), reduced ELAVL1, IRAK1 and NF-κB levels, while upregulating miR-146a-5p. In Silico analysis identified multiple putative molecular targets for miR-146a-5p, revealing strong interconnection among key targets, including ELAVL1, NF-κB, and IRAK1. These findings provide evidence to support the ameliorative role of BM-MSC-derived exosomes in mitigating DOX-induced AKI, associated with upregulation of miR-146a-5p and concurrent modulation of ELAVL1, IRAK1, and NF-κB, as well as NLRP3-associated inflammatory signaling, supporting the biological plausibility of a candidate miR-146a-5p/ELAVL1/IRAK1/NF-κB/NLRP3 regulatory network in AKI pathogenesis. Harnessing the reno-protective properties of exosomal therapy, as well as targeting the inflammasome-related signaling pathways, represents a compelling therapeutic paradigm warranting further mechanistic validation.

**Graphical Abstract:**

DOX induces oxidative stress and kidney damage, activating the NF-κB pathway and the NLRP3 inflammasome, which in turn facilitates caspase-1-mediated pyroptosis and the secretion of inflammatory cytokines (IL-1β and IL-18). Exosomes containing miR-146a-5p mitigate this process by targeting IRAK1 and ELAVL1, consequently inhibiting NF-κB signaling, diminishing inflammasome activation, and safeguarding renal cells from inflammation and pyroptotic injury. DAMPs: Damage-associated molecular patterns; DOX: Doxorubicin; ELAVL1: ELAV-like RNA-binding protein 1; EXO: Exosomes; GSDMD: Gasdermin D; IL-18: Interleukin-18; IL-1β: Interleukin-1 beta; IRAK1: Interleukin-1 receptor associated kinase 1; NF-κB: Nuclear factor-kappa B; NLRP3: Nucleotide-binding oligomerization domain-like receptor family pyrin domain containing 3; ROS: Reactive oxygen species; TLR: Toll-like receptor.

**Figure:**
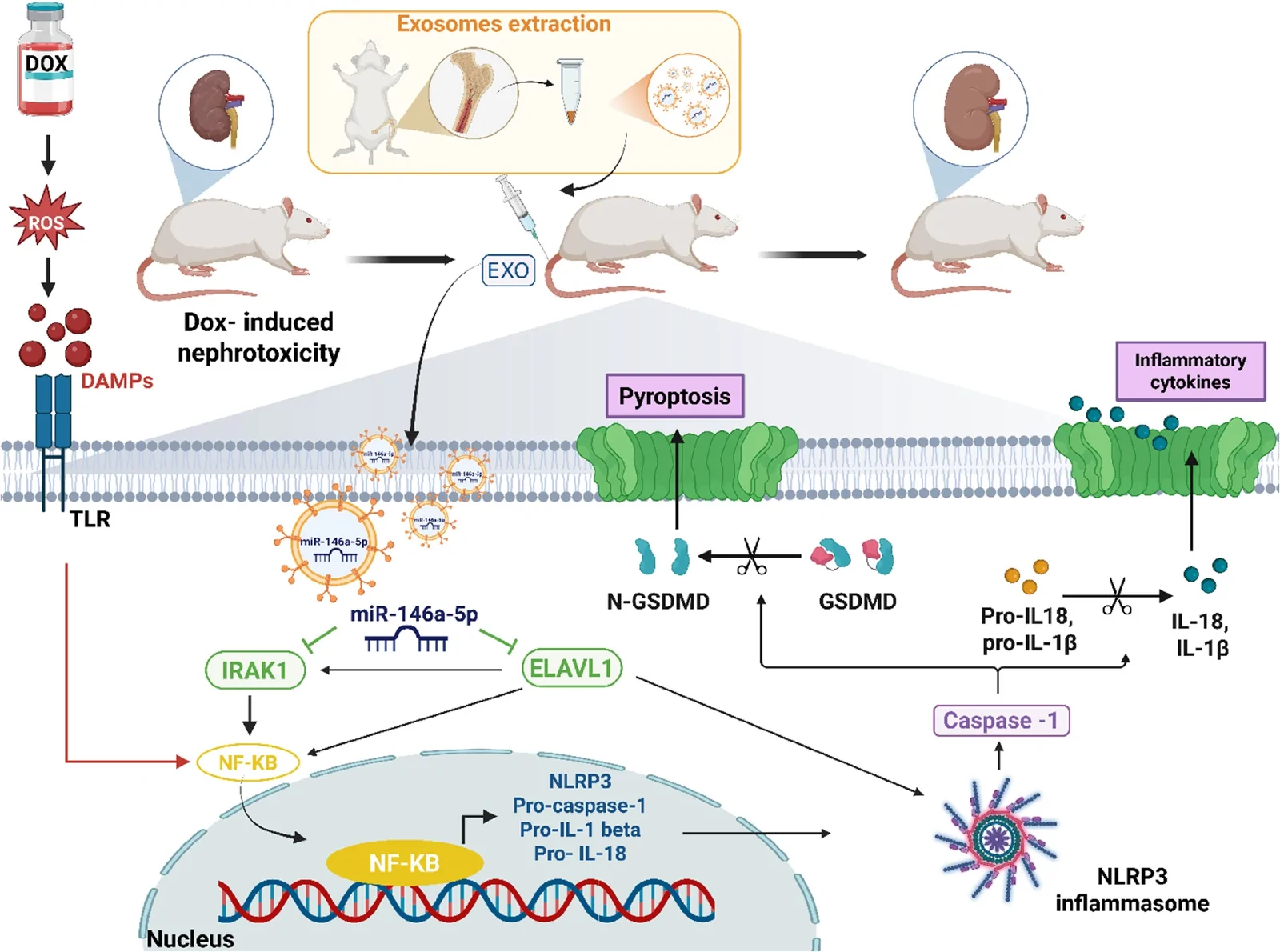

**Supplementary Information:**

The online version contains supplementary material available at https://doi.org/10.1186/s10020-026-01655-6.

## Introduction

Acute kidney injury (AKI) constitutes a significant global health burden associated with substantial morbidity, mortality, and healthcare expenditure. According to the International Society of Nephrology, 13.3 million patients are diagnosed with AKI annually, culminating in 1.7 million deaths globally (Han et al. 2024). AKI is characterized by an abrupt decline in renal function, arising from diverse etiological factors and underpinned by complex pathophysiological mechanisms. Besides, there is considerable risk of progression to chronic kidney disease (CKD) and end-stage renal disease, underscoring the importance of early diagnosis and timely intervention (Cao et al. 2025). The pathogenesis of AKI encompasses several factors, including sepsis (Wang et al. 2022), rhabdomyolysis (Matsushita et al. 2021) and nephrotoxic drugs (Zhang et al. 2020; Asaad et al. 2021).

AKI can be diagnosed using multiple biomarkers indicative of renal damage, such as neutrophil gelatinase-associated lipocalin (NGAL) (El-Ashmawy et al. 2022), kidney injury molecule-1 (KIM-1), and liver-type fatty acid-binding protein (L-FABP), in combination with conventional serum creatinine and urine output measurements (Bhatraju et al. 2020). KIM-1 is a transmembrane protein that is elevated in proximal tubular cells after injury, and increased urine KIM-1 is considered a potential early marker of tubular injury in AKI (Schrezenmeier et al. 2017). Current treatments for AKI mainly provide supportive care and focus on addressing complications instead of restoring damaged renal tissue, leading to consistently elevated morbidity and mortality rates. Therefore, there is an immediate need for innovative regenerative or cell-based therapies that can induce renal repair, restore kidney function, and improve outcomes (Ronco et al. 2019).

Doxorubicin (DOX), a nephrotoxic drug, is an anthracycline chemotherapeutic agent widely used to treat solid and hematologic malignancies, including lung, breast, ovarian, uterine, and cervical cancers, as well as leukemia (Antar et al. 2023). The anticancer activity of DOX is due to its ability to intercalate into the DNA helix and/or form covalent bonds with proteins involved in DNA replication and transcription, thereby inhibiting DNA, RNA, and protein synthesis, ultimately leading to cell death (Hussain et al. 2021). However, its clinical application is constrained by its adverse effects on several organs, particularly the heart and kidneys (Al Suleimani et al. 2023). The most common mechanism of DOX-induced nephrotoxicity is the excessive generation of reactive oxygen species (ROS) (Songbo et al. 2019). Besides, the accumulation of lipid peroxidation markers, such as malondialdehyde (MDA), and the reduction in antioxidant activity, such as catalase (CAT), lead to ROS stimulation of the apoptotic pathway in the kidney (Xiang et al. 2021; Soltani Hekmat et al. 2021). Inflammation also contributes significantly to DOX-induced kidney damage through the actions of local cytokines, including interleukin-1β (IL-1β), interleukin-18 (IL-18), and tumor necrosis factor-α (TNF-α) (Wu et al. 2021). Additionally, DOX induces nephrotoxicity by causing direct injury to the renal tubules and glomeruli (Kaymak et al. 2022).

Culminating evidence suggested the role of pyroptosis, a distinct form of programmed cell death, as one of the key pathways in DOX-induced nephrotoxicity (Li et al. 2025; Abd-Ellatif et al. 2022). Pyroptosis is currently recognized as a critical pathological determinant in the progression of a broad spectrum of renal disorders, encompassing AKI, renal interstitial fibrosis, diabetic nephropathy, and renal cell carcinoma (Xu et al. 2021; Deng et al. 2021; Zheng et al. 2022; Ding et al. 2023). Beyond the kidney, pyroptosis has been mechanistically implicated in the pathogenesis of neurodegenerative conditions, cardiovascular disorders, atherosclerosis, rheumatoid arthritis, infectious diseases, and malignancies, underscoring its significance as a potential therapeutic target for intervention in multiple diseases (Joshi et al. 2023; Zeng et al. 2020; Chang et al. 2020; Yuan et al. 2021; Zhang et al. 2022). Pyroptosis exhibits morphological features of both apoptosis and inflammatory necrosis, which is typically triggered by caspase-1-dependent inflammasomes and nucleotide-binding oligomerization domain-like receptor family pyrin domain containing 3 (NLRP3) (Eskander et al. 2025). The NLRP3 inflammasome complex can be stimulated *via* pathogen-associated molecular patterns (PAMPs) or damage-associated molecular patterns (DAMPs). Then the inflammasome triggers the activation of pro-caspase-1 to caspase-1(CASP-1), which breaks down pro-IL-1β and pro-IL-18 into active IL-1β and IL-18. Subsequently, these mature forms stimulate the immune response and cause inflammation (Eskander et al. 2025; Zhou et al. 2022). Caspase-1 also activates gasdermin-D (GSDMD) to its active form, which forms pores in the cell membrane (Dong et al. 2024). Activation of the NLRP3 inflammasome, followed by pyroptosis upregulation and cell membrane pore formation, was recently demonstrated in DOX-induced nephrotoxicity (Li et al. 2025; Abd-Ellatif et al. 2022).

ELAV-like RNA-binding protein 1 (ELAVL1) is a part of the pyroptosome that activates pyroptosis and promotes inflammatory cytokine secretion (Lv et al. 2023). Bei et al*.* highlighted that ELAVL1 regulates pyroptosis in cisplatin-induced AKI in mice (Bei et al. 2023). Moreover, interleukin-1 receptor associated kinase 1 (IRAK1), an intracellular kinase, upregulates the immune response and activates both nuclear factor kappa B (NF-κB) and mitogen-activated protein kinase (MAPK) pathways. The activation of NF-κB signaling and the NLRP3 inflammasome promotes pyroptosis and contributes to the development of diabetic nephropathy (Li et al. 2024). Nevertheless, the role of ELAVL1 and IRAK1 in the pathogenesis of DOX-induced AKI has not been adequately studied.

Mesenchymal stem cells (MSCs) show critical characteristics of self-renewal, differentiation, and immunomodulation. These cells play a vital role in regenerative medicine as they can generate a favorable environment for damaged tissues healing (Cao et al. 2021). Bone marrow-derived mesenchymal stem cells (BM-MSCs) are multipotent progenitor cells that have been extensively studied for their ability to repair and regenerate tissues and modulate the immune system (Liu et al. 2023). MSCs facilitate the repair of cardiovascular, pulmonary, and spinal cord injuries, as well as the repair of autoimmune disorders, liver, bone, and cartilage diseases (Saeedi et al. 2019; Huang et al. 2016; Al-Sherief et al. 2025). Previous studies have demonstrated that the administration of exogenous BM-MSCs can alleviate AKI and enhance renal function by targeting multiple pathways, including mitophagy, apoptosis, and renal fibrosis (Guo et al. 2021; Zhu et al. 2019; Wang et al. 2023; Shahin et al. 2026).

Exosomes (EXO) are small vesicles involved in cellular communication and the transfer of proteins and nucleic acids (Chen et al. 2024). They have an important role as a diagnostic and prognostic tool in several diseases (Zou et al. 2023; Aheget et al. 2020). Exosomes have been studied as biomarkers for diagnosing kidney disorders (Awdishu et al. 2021). The therapeutic potential of exosomes has been demonstrated in different diseases, such as bone (Jin et al. 2021), liver (Rong et al. 2019; El-Derany and AbdelHamid 2021), neurological (Zhuang et al. 2022), and cardiovascular disorders (El-Derany and AbdelHamid 2022; Wang et al. 2018). Furthermore, there is increasing evidence underscoring the significant beneficial effect of exosomes in many kidney diseases, including nephrotic syndrome (Jia et al. 2024), AKI (Jin et al. 2023), and CKD (Alasmari et al. 2022). Exosomal cargo includes a wide range of proteins, such as enzymes, transcription factors, receptors, extracellular matrix proteins, lipids, and nucleic acids including microRNAs (miRNA), DNA, and mRNA (Skotland et al. 2020). MiRNAs are single-stranded RNA molecules, approximately 22 nucleotides in length (Bushati and Cohen 2007), which are crucial in various cellular processes, including cell differentiation, proliferation, migration, and programmed cell death (Ghafouri-Fard et al. 2020; Vafadar et al. 2019). The dysregulation in their expression is linked to different diseases, such as diabetes, cardiovascular disease, neurological impairment, and cancer (Hosseinahli et al. 2018). MiR-146a-5p is one of the exosomal miRNAs (Huang et al. 2022; Song et al. 2017) that has been shown to exert an anti-inflammatory effect in different diseases through targeting tumor necrosis factor receptor-associated factor-6 (TRAF6) (Hua et al. 2022), NLRP3 (Yang et al. 2022), and nuclear factor erythroid 2-related factor 2 (Nrf2) (Rasoulinejad et al. 2021).

The mounting scientific evidence demonstrating pyroptosis involvement in renal disease pathogenesis suggests that targeting of pyroptotic mechanisms represents a rational and promising therapeutic strategy for attenuating the excessive inflammatory responses that characterize AKI. Despite growing interest in exosome biology, several knowledge gaps persist regarding their precise mechanistic contributions to AKI. The current study aimed to delineate the attenuative effect of BM-MSCs-derived exosomes in DOX-induced AKI in an experimental rat model through the investigation of oxidative stress markers (MDA and CAT activity), inflammatory cytokines (IL-1β and IL-18), markers of the pyroptosis pathway (NLRP3, caspase-1, and GSDMD), as well as miR-146a-5p, ELAVL1 and IRAK1 expression.

## Materials and methods

### In silico database analysis

#### Selection of miRNA

The selection of miR-146a-5p as a target in the current study was based on previous studies and in silico bioinformatic evidence. A previous study showed that pretreatment with exosomes derived from IL-1β-primed MSCs rich in miR-146a enhanced survival and decreased inflammation in a rat sepsis model (Song et al. 2017). The localization of miR-146a-5p inside BM-MSCs-derived exosomes was then confirmed bioinformatically using the RNA-LOCATE website http://www.rnalocate.org (Accessed in February 2023, then revised in January 2026) (Wu et al. 2025).

#### Determination of different targets of miR-146a-5p and AKI.

In order to identify different targets of miR-146a-5p, three in silico tools were deployed: TargetScan https://www.targetscan.org (Ritchie 2016), miRDB https://www.mirdb.org (Chen and Wang 2020), and DIANA tools http://WWW.diana.imis.athena-innovation.gr/DianaTools/index.php (Vlachos et al. 2015) (Accessed in February 2023, then revised in January 2026). The different targets of AKI were identified from five databases: GeneCards database https://www.genecards.org/search/results?q=AKI (Safran 2010), The Open Targets Platform database https://platform.opentargets.org/disease/MONDO_0002492/associations (Carvalho-Silva et al. 2019), Comparative Toxicogenomics Database (CTD) https://ctdbase.org/detail.go?type=disease&acc=D058186 (Davis et al. 2023), MalaCards database https://www.malacards.org/card/acute_kidney_failure?search=AKI (Rappaport et al. 2017), and DisGeNET database https://disgenet.com/search?view=DISEASES&idents=C2609414&source=ALL&tab=GDA (Piñero et al. 2020) (Accessed in September 2026). The data were analyzed using the Jvenn tool (https://jvenn.toulouse.inrae.fr/app/example.html) to produce a Venn diagram, and the common targets between miR-146a-5p and AKI were identified (Bardou et al. 2014).

#### Protein–protein interaction (PPI), drug-target networks, and functional enrichment analysis

The PPI network of the common targets was constructed by uploading the relevant targets to the STRING database Version 12.0 (https://string-db.org/) (Szklarczyk et al. 2023) (Accessed in September 2026), using a default confidence level of > 0.4. The network visualization was performed using the Cytoscape 3.10.4 software, developed by NIGMS in the United States (Shannon et al. 2003) (Accessed in September 2026). Then, the common targets were filtered by degree value and subsequently, ranked based on betweenness and closeness by using Cytoscape plugin CytoNCA (Tang et al. 2015). The top 20-ranked proteins were identified as hub targets. The Kyoto Encyclopedia of Genes and Genomes (KEGGs) pathway enrichments were conducted using the ShinyGO software https://bioinformatics.sdstate.edu/go/#tab-6415-3 (Ge et al. 2020) (Accessed in September 2026). After that, the PPI network of the pyroptosis-related targets and three targets demonstrating the highest functional relevance to the pyroptotic signaling (ELAVL1, IRAK1, and NF-κB) was constructed by the STRING database Version 12.0 (https://string-db.org/) (Szklarczyk et al. 2023) (Accessed in August 2026), with a default confidence level of > 0.4.

#### The functional distribution

The functional distribution of the common targets through different pathways was evaluated using the Panther Database (https://www.pantherdb.org) (Mi et al. 2021).

#### Binding interactions

The binding interaction between miR-146a-5p with ELAVL1 and IRAK1 was studied using the RNA22 v2 microRNA target detection database (https://cm.jefferson.edu/rna22/Interactive/), Jefferson Computational Medicine Center (Miranda et al. 2006) (Accessed in February 2023, then revised in January 2026) after inserting the FASTA format of each obtained from different databases. [FASTA format of the mature rno-miR146a-5p retrieved through miRbase Reference Sequence: https://mirbase.org/mature/MIMAT0017132. FASTA format of ELAVL1 mRNA transcript retrieved through Ensembl Reference Sequence: https://www.ensembl.org/Rattus_norvegicus/Transcript/Exons?db=core;g=ENSRNOG00000001069;r=12:7441699-7482631;t=ENSRNOT00000001415 and IRAK1 mRNA Ensembl Reference Sequence: https://www.ensembl.org/Rattus_norvegicus/Transcript/Exons?db=core;g=ENSRNOG00000060869;r=X:156920081-156929825;t=ENSRNOT00000092207].

Finally, to further explore ELAVL1's post-transcriptional regulatory role, RNA-binding protein (RBP) target interactions were examined using the ENCORI/starBase database (https://rnasysu.com/encori/) (Li et al. 2014) (Accessed in August 2026), which integrates CLIP-seq data to identify experimentally supported RBP binding sites on target transcripts. ELAVL1 was selected as the RBP of interest, and the analysis was queried against the pyroptosis pathway-related targets identified in the present study (IRAK1, NLRP3, CASP1, GSDMD, IL1β, IL18, and NF-κB) to identify transcripts with experimentally supported ELAVL1 binding.

### Isolation and characterization of BM-MSCs-derived exosomes

#### Isolation of BM-MSCs-derived exosomes

Isolation of BM-MSCs-derived exosomes was performed according to a previous study (Wan et al. 2022). Briefly, BM-MSCs reached the third-fifth passage with an 80% confluence. They were collected and washed three times with phosphate-buffered saline (PBS). BM-MSCs were incubated in a medium free of extracellular vesicles (EVs) for 48 h. Subsequently, 2 mL of cell culture supernatant was collected and centrifuged at 4 °C, with forces of 500 × g and 2000 × g applied for 15 min each to remove cells, cell debris, and apoptotic bodies. The supernatant was centrifuged at 10,000 × g for 20 min. After centrifugation, the supernatant was collected and filtered using a 0.22 μm filter membrane. The EVs were isolated by ultracentrifugation at 100,000 × g for 70 min. The precipitate was then reconstituted in 1 × PBS and centrifuged at 100,000 × g for 70 min to remove any impurities from the proteins. Ultimately, the precipitate was resuspended in 1 × PBS and stored at −80 °C.

#### Characterization of BM-MSCs-derived exosomes

The characterization of the isolated exosomes was performed according to the guidelines of the International Society for Extracellular Vesicles (Théry et al. 2018). Exosomes were morphologically characterized using transmission electron microscope (TEM) (Rikkert et al. 2019), then the surface markers were detected using flow cytometry (Li et al. 2017; Bian et al. 2014).

##### Transmission electron microscope (TEM)

TEM characterization was performed according to a previous study (Rikkert et al. 2019). Briefly, 10 μL of exosomes were applied to carbon-coated 220-mesh copper grids and incubated for 2 min. Adherent exosomes were negatively stained with 1% phosphotungstic acid (Sigma-Aldrich, St Louis, MO, USA). The stain was allowed to dry at room temperature for 1 h. Ultimately, the exosomes were examined using TEM utilizing a JEM-1010 instrument (Manufactured by JEOL Ltd., Tokyo, Japan). The acceleration voltage used was 70 kV. Dynamic light scattering (DLS) was performed using a Zetasizer Nano ZS (Malvern Panalytical, UK) at 25 °C to determine particle size distribution and the polydispersity index, as previously described (Instruments 2004).

##### Flow cytometry

Flow cytometry was performed initially to characterize MSC immunophenotype using a BD FACS flow cytometer. Cells were washed twice with PBS. Approximately 1 × 10^5^ cells were resuspended in 100 µL of staining buffer (PBS augmented with 1% bovine serum albumin). Cells were treated with fluorochrome-conjugated monoclonal antibodies targeting CD105 and CD45 for 30 min in the dark at 4 °C for surface marker analysis. Unstained cells were utilized as negative controls to establish baseline fluorescence and gating parameters. Following incubation, cells were rinsed twice with PBS and resuspended in 500 µL of buffer for acquisition. Data were analyzed using FlowJo software.

Additionally, exosomal surface markers were identified by flow cytometry using antibodies against CD63, CD9, and CD45 (Theodoraki et al. 2021). Briefly, A concentration of 1 × 10^10/ml of exosomes was suspended in 100 µL of filtered PBS, containing 2% exosome-depleted fetal bovine serum (FBS), treated with protease and phosphatase inhibitors, and subsequently stained with a panel of three primary antibodies. Antibodies, with a dilution of 1:100, used for exosomes monoclonal staining were purchased from ThermoFisher Scientific, USA (Cat. No. MEM-259), (Cat. No. MM2/57), and (Cat. No. 30-F11), respectively. To stain exosomes, 15 µL of exosome solution was mixed with 5 µL of diluted antibody (1:100) and incubated for 45 min in the dark at room temperature. Additionally, the exosomes were rinsed with a washing buffer consisting of 0.2 µm-filtered PBS and 2% Exosome-depleted FBS from Thermo Fisher, USA. Data analysis was conducted with the BE14548 program, Version: Navios EX program. Positive exosome counts were determined as the ratio of EVs positive for each biomarker to the total EVs recorded using anti-CD9, CD45, and CD63 labeling.

### Animals and chemicals

Twenty-four Sprague Dawley rats, weighing 150–250 g, were purchased from The National Cancer Institute in Cairo, Egypt. DOX was purchased as Doxorubicin Hexal® (Hexal AG, Holzkirchen, Germany). The experimental protocol was carried out in accordance with the Guide for Care and Use of Laboratory Animals published by the US National Institutes of Health (NIH Publication No. 85–23, revised 2011) (Council et al. 2011). The Research Ethics Committee at the Faculty of Pharmacy, Ain Shams University, Egypt (Approval no. 228, 2023) has approved the experimental protocol.

### Experimental design

**Fig. 1:**
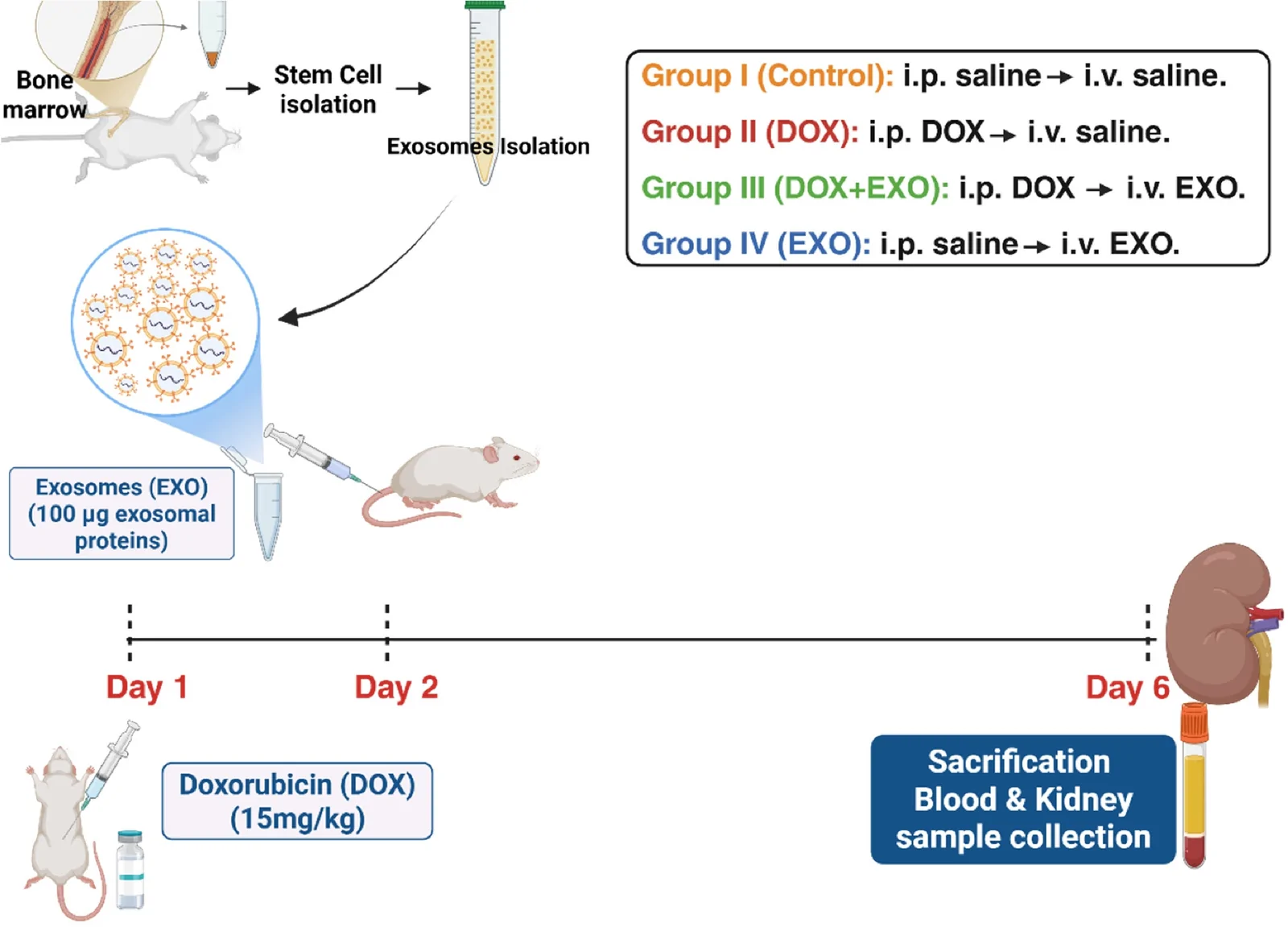
The Experimental Design of the Study. DOX, doxorubicin; EXO, exosomes

Rats were acclimatized for one week, during which they were allowed free access to food and water, housed in open cages and underwent a 12-h cycle of day and night at a temperature of 25 °C. Then rats were divided into four groups (n = 6), as illustrated in Fig. 1. The first group was the control group (Control), which received an intraperitoneal (*i.p.*) injection of normal saline on day one, followed by an intravenous (*i.v*.) injection of normal saline after 24 h. The second group was the diseased group (DOX); rats received a single *i.p.* injection of DOX (15 mg/kg) on day one, then after 24 h, they received an *i.v.* injection of normal saline. The dose of DOX and its nephrotoxicity model were chosen in accordance with previous studies (El-Sheikh et al. 2012; Rafiee et al. 2020) and further confirmed by a pilot dose-ranging study. Three doses of DOX (10, 15, and 20 mg/kg) were tested. The 10 mg/kg dose produced weak renal injury, whereas the 20 mg/kg dose caused marked toxicity with increased mortality. Consequently, the 15 mg/kg dose was chosen as it consistently induced moderate nephrotoxicity with acceptable survival. The third group (DOX + EXO) received DOX (15 mg/kg, *i.p*.) on day one, then, after 24 h, received an *i.v.* injection of exosomes (100 μg per rat). The fourth group (EXO) received exosomes only; rats received an *i.p.* injection of normal saline on day one, then after 24 h, they received an *i.v.* injection of exosomes (100 μg per rat) (Jin et al. 2023; Oyar et al. 2022).

On the 6th day, blood samples were collected from the orbital sinus region after anesthesia using sodium pentobarbital (60 mg/kg, *i.p.*) (Abd-Ellatif et al. 2022). Blood samples were left to coagulate, then centrifuged for 20 min at 3000 rpm at 4 ^◦^C to obtain serum samples, then immediately stored at −80 ^◦^C. Rats were sacrificed by cervical dislocation. The two kidneys were immediately collected and washed using cold 0.9% saline, then dried and weighed to calculate the renal somatic index (RSI). Calculation was performed by dividing the kidney's weight (g) by the rat's weight (g) and multiplying by 100. Each right kidney was preserved in 10% neutral buffered formalin (NBF) for histopathological and immunohistochemical analyses. The left kidneys were cut longitudinally into two halves; the first half was stored in −80 ^◦^C in PBS for biochemical analysis, while the other half was stored in RNAlater® (Sigma-Aldrich, St Louis, MO, USA) for further gene expression analysis. Tissue homogenate was prepared by grinding the frozen kidney sections in cold PBS, then centrifuging it for 20 min at 4 ^◦^C at 3000 rpm. Then, the supernatant was preserved at −80 ^◦^C for the following investigations. All biomarkers measured in tissue homogenate were normalized to tissue protein content (mg protein).

### Histopathological examination

After sample collection, they were stored and preserved in 10% NBF solution, then cut down, rinsed with water, dehydrated using ascending concentrations of alcohol, cleaned in xylene, and embedded in paraffin. Staining with Hematoxylin & Eosin (H&E) for histological scoring was performed on thin sections of 4–6 µm (Suvarna et al. 2018). Prepared slide sections were examined by a light digital microscope (Olympus xc30, Tokyo, Japan). A grading system scaled from 0 to 3 was employed, where (0) reflects normal histological structure; 1 = kidney damage in less than 25% of tissue; 2 = kidney damage in 25%–50% of tissue, and 3 = kidney damage affecting > 50% of tissue. Kidney damage levels were scored by the pathologist according to focal glomerular necrosis, Bowman capsule dilatation, tubular epithelial degeneration, tubular epithelial necrosis, tubular dilatation, interstitial inflammation, infiltration, and congestion in all sections (Colbay et al. 2010).

In the second examination, different parameters were scored according to dilation in Bowman's cavity, vacuolization in tubule epithelial cells, degeneration, accumulation of intratubular hyaline, and vasocongestion in all sections (Sancak et al. 2016). As a result of the examinations, it was scored according to the following scale: 0 = normal kidney (no damage); 1 = minimal damage (< 25% damage); 2 = slight damage (25–50% damage); 3 = medium damage (50–75% damage); and 4 = severe damage (> 75% damage).

### Assessment of kidney function

Serum creatinine (Scr) and blood urea nitrogen (BUN) were used as indicators of kidney function. Both Scr and BUN were determined using colorimetric assays with the available commercial kits provided by Spectrum Diagnostics, Cairo, Egypt (Cat. No. 234 001 and 318 001), respectively, following the manufacturer's instructions. Scr and BUN are represented in the form of mg/dl.

### Assessment of renal oxidative stress

Renal oxidative stress was assessed by measuring CAT activity to determine kidney antioxidant activity and MDA levels as a marker of lipid peroxidation. CAT activity was determined in tissue homogenate colorimetrically using the commercial kit provided by Elabscience, USA (Cat. No. E-BC-K031-M), in accordance with the manufacturer's instructions. Briefly, CAT activity was quantified by allowing the enzyme to decompose hydrogen peroxide (H₂O₂) and then rapidly stopping the reaction with ammonium molybdate. The remaining H₂O₂ forms a yellow complex with ammonium molybdate, and the intensity of this color at 405 nm indicates the catalase activity, expressed as U/mg protein.

MDA was determined in tissue homogenate colorimetrically using the commercial kit provided by Abcam (Colorimetric/Fluorometric) (Cat. No. ab118970), referenced by the manufacturer's instructions. MDA reacts with thiobarbituric acid (TBA) in acidic media at 95 °C for 30 min to form a pink product, thiobarbituric acid reactive. The absorbance of the product was measured at 534 nm. MDA concentration is expressed as nmol/mg protein.

### Enzyme-linked immunosorbent assays (ELISA)

KIM-1 was determined in tissue homogenate using the rat KIM-1 ELISA Kit, MyBioSource, USA (Cat. No. MBS2702467) according to the kit's instructions, and the concentration was expressed as pg/mg protein. The renal IL-1β & IL-18 levels were determined in tissue homogenate using the rat ELISA kits, Elabscience, USA (Cat. No. E-EL-R0012) and BT lab, China (Cat. No. E0117Ra), respectively. GSDMD, a specific biomarker of the pyroptosis pathway, was measured in tissue homogenate using the rat ELISA kit (BT Lab, China; Cat No: E2451Ra), and results were represented as ng/mg protein. Additionally, renal IRAK1 (Cat. No. MBS076462), ELAVL1 (Cat No. MBS2019259), and NF-κB (Cat. No. MBS453975) were measured in tissue homogenate using rat ELISA kits, provided by MyBioSource, USA.

The procedure of each test was referenced by each kit's instructions. Briefly, a sandwich ELISA was performed in which the target antigen was captured between a plate-bound antibody and a biotinylated detection antibody, followed by binding of a streptavidin–enzyme conjugate, with tetramethylbenzidine (TMB) as the chromogenic substrate. Then, a stop solution was used to stop the reaction for accurate colorimetric measurement. The color intensity was quantified at 450 nm using a microplate reader (ChroMate-4300, FL, USA).

### Immunohistochemical (IHC) analysis of NLRP3 and Caspase-1

Renal expressions of NLRP3 and caspase-1 were evaluated using IHC analysis. Briefly, paraffin-embedded renal sections were dewaxed and rehydrated, then subjected to heat-induced antigen retrieval. Sections were then incubated with rabbit polyclonal primary antibodies against caspase-1 (ABclonal, Cat. No. A18646; 1:100) and NLRP3 (Servicebio, Cat. No. GB114320; 1:300). After washing, sections were incubated with the appropriate biotinylated secondary antibody, and the signal was amplified using the avidin–biotin–peroxidase complex (ABC) method (VECTASTAIN ABC-HRP kit, Vector Laboratories). Immunoreactivity was visualized using 3,3′-diaminobenzidine (DAB, Cat. No. D7304, Sigma-Aldrich) as the chromogen. The stained sections were examined using an Olympus microscope (BX-63). Two main criteria, the color intensity and area percentage (%) of positively stained cells, were basically used for this assessment by using Fiji Image J analysis. For color intensity, a grading system scaled from 0 to 3 was used, in which grade 0 = no staining, grade 1 = weak staining, grade 2 = moderate staining, and grade 3 = strong staining (Schindelin et al. 2012).

### Total RNA extraction from renal tissue

RNA extraction was performed using TRIzol reagent (Cat. No. 15596026; Life Technologies, USA) according to the manufacturer’s instructions. Briefly, after homogenizing the sample with TRIzol Reagent, chloroform was added, and RNA was precipitated from the aqueous phase using isopropanol. Afterward, the RNA pellet was washed with 75% ethanol, then centrifuged at 7,500 × g for 5 min at 4 °C. The supernatant was removed, and the pellet was air-dried for 5 to 10 min. Finally, the pellet was dissolved in RNase-free water and incubated at 55–60 °C for 10 min to ensure complete solubilization. Afterwards, RNA purity and concentration were measured using a NanoDrop spectrophotometer (Thermo Scientific, USA).

### Complementary DNA (cDNA) synthesis and quantitative real-time polymerase chain reaction (qRT-PCR) of miR-146a-5p, IRAK1, and ELAVL1

Complementary DNA (cDNA) was synthesized from single-stranded RNA using the QuantiTect Reverse Transcription Kit (Qiagen, USA, Cat. No. 205311). cDNA was produced from 1 µg of total RNA using the QuantiTect Reverse Transcription Kit (Qiagen, USA) according to the manufacturer's protocol, which included genomic DNA removal and reverse transcription at 42 °C for 15 min, followed by enzyme inactivation at 95 °C for 3 min.

Quantitative real-time PCR (qRT-PCR) was performed using Maxima SYBR Green/Fluorescein qPCR Master Mix (2 ×) (Cat. No. K0241) on a Rotor-Gene Q real-time PCR system (Qiagen, USA). Thermal cycling conditions consisted of an initial denaturation at 95 °C for 10 min, followed by 45 cycles of denaturation at 95 °C for 10 s, annealing at 60 °C for 15 s, and extension at 72 °C for 15 s, as previously described (El-Dessouki et al. 2025). A melting curve analysis was performed from 72 °C to 95 °C to confirm amplification specificity. Relative expression levels of miR-146a-5p, IRAK1, and ELAVL1 were calculated using the 2⁻ΔΔCt method (Livak and Schmittgen 2001). GAPDH was used as the internal housekeeping gene for mRNA normalization, while U6 small nuclear RNA (U6 snRNA) was used as the endogenous control for miRNA expression analysis. The primer sequences used in this study are listed in Table 1.

**Table 1:** The primers of miR-146a-5p, IRAK1, ELAVL1, GAPDH, and U6 used for RT-qPCR

Gene name	Forward (5′- 3′)	Reverse (5′–3′)
miR-146a-5p	CGCGTGAGAACTGAATTCCA	AGTGCAGGGTCCGAGGTATT
IRAK1	GCTGTGGACACCGAT	ACACCTACCCACATCG
ELAVL1	CGCAGAGATTCAGGTTCTCC	CCAAACCCTTTGCACTTGTT
GAPDH	GAAGGTGGTGAAGCAGGCATC	GTGGGAGTTGCTGTT GAAGTC
U6	CTCGCTTCGGCAGCACA	AACGCTTCACGAATTTGCGT

### Statistical analysis

GraphPad Prism V 8.0.1 (GraphPad Software Inc., San Diego, CA, USA) was used for statistical analysis and graphical representation. The results were analyzed using one-way analysis of variance (ANOVA), followed by Tukey's post hoc test. The data were shown in the form of mean ± standard error of the mean (mean ± SEM). Statistical significance was set at a *p*-value < 0.05.

## Results

### In silico and bioinformatics analysis

**Fig. 2:**
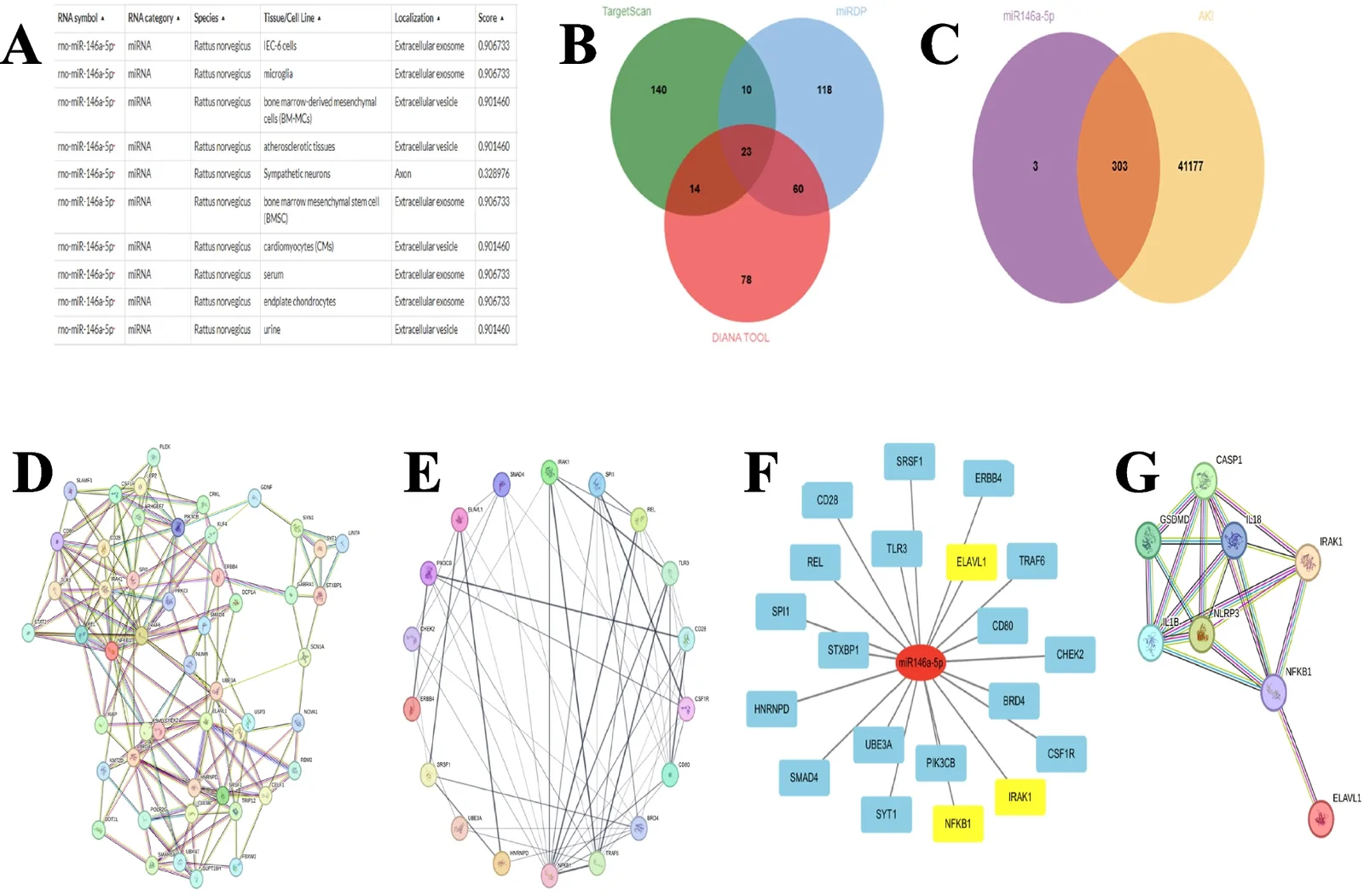
In Silico miR-146a-5p Analysis and Target Prediction. **A** Prediction of subcellular locations of miR-146a-5p through RNA-LOCATE website (http://www.rnalocate.org/). **B** Venn diagram created using Jvenn tool (https://jvenn.toulouse.inrae.fr/app/example.html) of the predicted miR-146a-5p targets through TargetScan (https://www.targetscan.org/vert_80/), miRDB (https://mirdb.org/) and DIANA TOOLS (https://dianalab.e-ce.uth.gr/html/dianauniverse/index.php?r=MicroT_CDS/index). **C** Venn diagram created using Jvenn tool (https://jvenn.toulouse.inrae.fr/app/example.html) of the predicted common targets between miR-146a-5p and AKI. **D** Protein–Protein Interactions (PPI) network through STRING database Version 12.0 (https://string-db.org/) of the common 303 targets. **E** Protein–Protein Interactions (PPI) network through STRING database Version 12.0 (https://string-db.org/), the network visualization was performed using the Cytoscape 3.10.4 software. **F** miR-146a-5p -targets network, Diamond: miR-146a-5p, and Ellipse: 20 hub targets (yellow are our measured targets in this study). **G** Protein–Protein Interactions (PPI) network through STRING database Version 12.0 (https://string-db.org/) for pyroptosis-related targets. The interaction score was established at 0.4, indicating medium confidence and serving as the default level

The results compiled from the RNA-LOCATE website confirmed the expression of miR-146a-5p inside BM-MSCs-derived vesicles and exosomes (Fig. 2A). The respective targets of miR-146a-5p obtained from different databases (TargetScan, miRDB, and DIANA TOOLS) were found to be 187, 211, and 175 targets, respectively, as shown in the Venn diagram (Fig. 2B and Supplementary Table 1). Besides, the AKI targets obtained from five databases (GeneCards database, The Open Targets Platform database, CTD Database, MalaCards database, and DisGeNET database) were 10,365, 1456, 41,357, 574, and 114, respectively (Supplementary Table 2). After removing duplicate entries, the total number of targets was reduced to 41,480 (Supplementary Table 3). Using the Jvenn tool, 303 common targets between the miR-146a-5p and AKI were obtained (Fig. 2C).

A PPI diagram was created using 303 overlapped targets in the STRING database to visualize the interactions among them. After excluding the unconnected nodes, the PPI network consisted of 51 nodes and 177 edges (Fig. 2D). The Cytoscape software 3.10.4 was used to integrate this network for further analysis. The CytoNCA app was used to rank the 51 targets based on Betweenness and Closeness after filtering by the degree value (Supplementary Table 4). Then, the 20 common targets were input into Cytoscape to construct a drug-targets network (Fig. 2E). In addition, a PPI network of the top 20 hub targets (NFKB1, BRD4, TRAF6, ELAVL1, SRSF1, CSF1R, REL, SMAD4, PIK3CB, CD80, CHEK2, ERBB4, SPI1, STXBP1, UBE3A, HNRNPD, SYT1, TLR3, CD28, IRAK1) with miR-146a-5p was created (Fig. 2F). STRING PPI network analysis showed that the pyroptosis-related targets (CASP1, GSDMD, IL18, IL1β, NLRP3, IRAK1, NF-κB) form a closely interconnected functional cluster, consistent with their established roles in inflammasome activation. ELAVL1 was linked to this core network through a direct interaction with NF-κB (Fig. 2G and Supplementary Table 5).

**Fig. 3:**
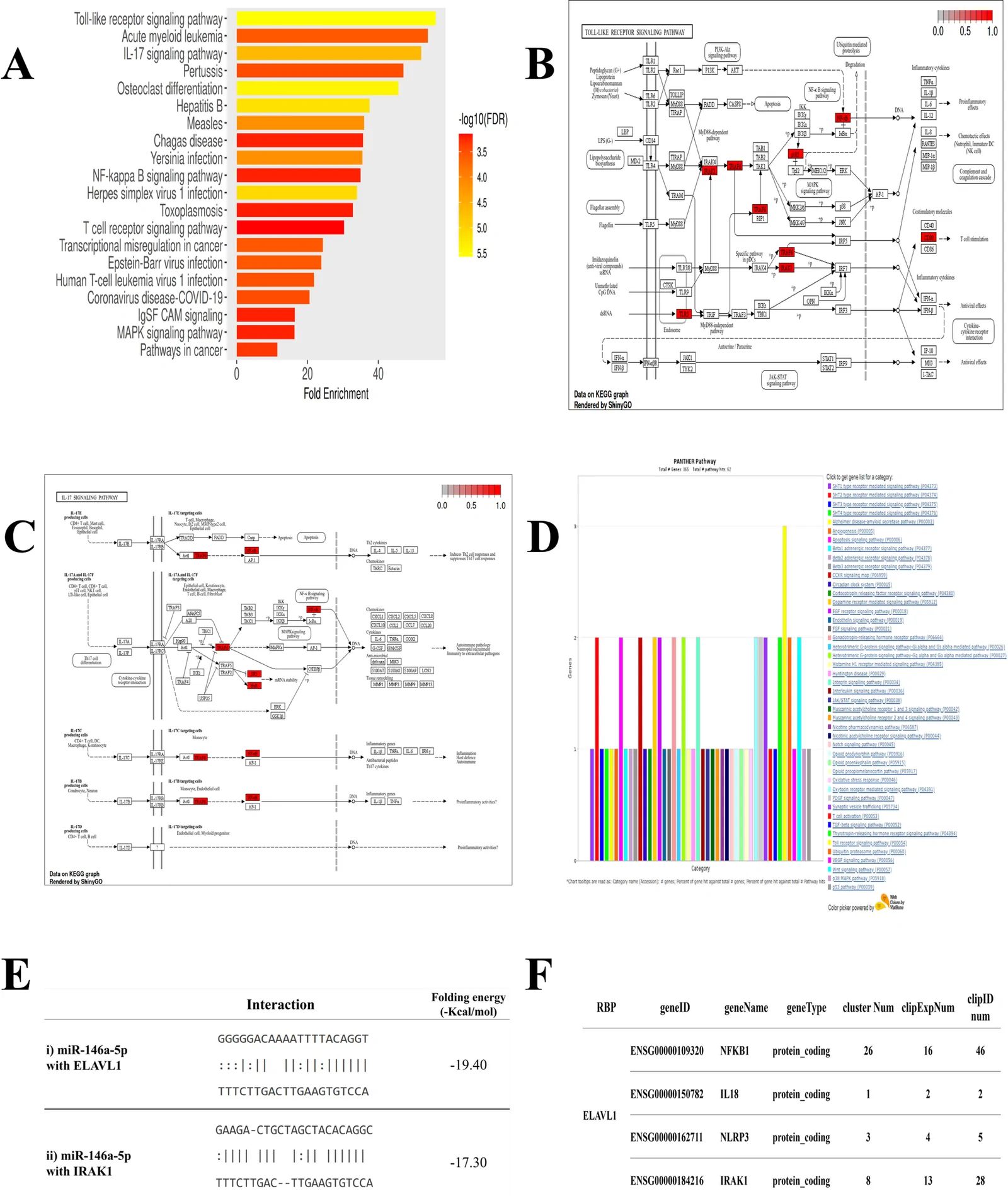
Pathway enrichment analysis, binding interaction prediction, and post-transcriptional regulatory network of miR-146a-5p target genes. **A** The top 20 significantly enriched Kyoto Encyclopedia of Genes and Genomes (KEGG) pathway (sorted by Fold Enrichment), **B **Toll-like receptor signaling pathway, **C** IL-17 signaling pathway, **D** The functional distribution of these genes through different pathways was done using Panther Database (https://www.pantherdb.org/). **E (i)** The predicted binding sequences of miR-146a-5p with ELAVL1,** (ii)** The predicted binding sequences of miR-146a-5p with IRAK1, using the database RNA22 v2 microRNA target detection (https://cm.jefferson.edu/rna22/Interactive/). Jefferson Computational Medicine Center after inserting the FASTA format of each [FASTA format of the mature rno-miR146a-5p obtained through miRbase Reference Sequence: https://mirbase.org/mature/MIMAT0017132. FASTA format of ELAVL1 mRNA transcript Ensembl Reference Sequence: https://www.ensembl.org/Rattus_norvegicus/Transcript/Exons?db=core;g=ENSRNOG00000001069;r=12:7441699-7482631;t=ENSRNOT00000001415 and IRAK1 mRNA Ensembl Reference Sequence: https://www.ensembl.org/Rattus_norvegicus/Transcript/Exons?db=core;g=ENSRNOG00000060869;r=X:156920081-156929825;t=ENSRNOT00000092207]. Databases were accessed in February 2023, then revised in January 2026. **F** ELAVL1 binding sites on pyroptosis pathway-related target transcripts (NF-κB, IL18, NLRP3, IRAK1) were identified using the ENCORI/starBase database (https://rnasysu.com/encori/)

The 20 common targets were submitted to the ShinyGO analysis tool for KEGG Pathways to validate the key functional and biological properties of the relevant proteins. KEGG Pathways with a *P* value < 0.05 included the Toll-like receptor (TLR) signaling pathway and the IL-17 signaling pathway are among the top two pathways (Fig. 3A, B, C). Besides, the functional distribution of these targets also highlighted that oxidative stress response and TLR signaling pathways were significantly enriched (Fig. 3D). miR-146a-5p showed binding interaction with ELAVL1 at folding energy – 19.4 kcal/mol (Fig. 3E (i)) and with IRAK1 at folding energy – 17.30 kcal/mol (Fig. 3E (ii)), as predicted *via* the database RNA22 v2 microRNA target detection by Jefferson Computational Medicine Center https://cm.jefferson.edu/rna22/Interactive/. Finally, ENCORI/starBase analysis identified experimentally supported ELAVL1 binding sites within the NF-kB, IL18, NLRP3, and IRAK1 transcripts among the queried pyroptosis pathway targets, with cluster numbers of 26, 1, 3, and 8 and CLIP experiment support numbers of 16, 2, 4, and 13, respectively (Fig. 3F and Supplementary Table 6).

### Characterization and identification of BM-MSCs-derived exosomes

**Fig. 4:**
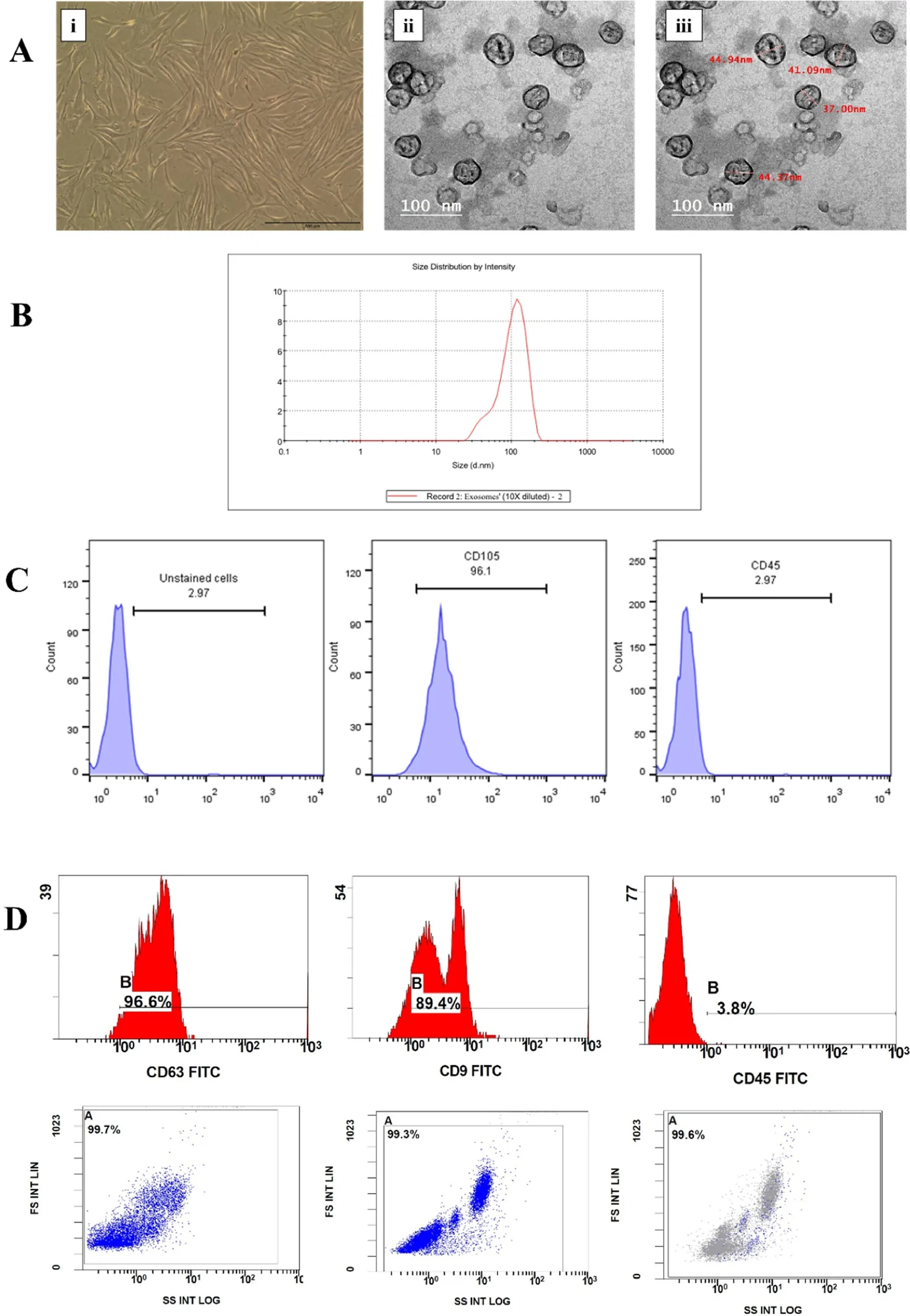
Characterization and Identification of BM-MSCs and BM-MSCs-derived Exosomes. **A (i)** Light micrograph of the spindle-shaped morphology of MSCs. **(ii)** TEM picture of the spherical-shaped BM-MSCs-derived exosomes. **(iii)** TEM picture of the size of BM-MSCs-derived Exosomes. **B** Size analyses of BM-MSCs-derived exosomes: dynamic light scattering (DLS) of exosomes. **C** Flow cytometry results of BM-MSCs surface markers (CD105 and CD45). **D** Flow cytometry results and dot plot of exosome surface markers (CD63, CD9, and CD45). BM-MSCs: Bone marrow-derived mesenchymal stem cells; TEM: Transmission electron microscope

BM-MSCs and their derived exosomes were characterized using TEM, which showed that BM-MSCs had a spindle morphology, and exosomal particles were spherical vesicles (Fig. 4A (i), (ii), (iii)) with a diameter of 30–150 nm as the results of DLS (Fig. 4B). The results of polydispersity index using Zetasizer Nano ZS equals 0.353, indicating the moderate polydispersity. In addition, flow cytometry analysis showed that BM-MSCs were positive for the surface marker CD105 and negative for CD45. On the other hand, exosomes were positive for CD63 and CD9 and negative for CD45, indicating pure exosomal isolation (Fig. 4C & D).

### The Modulatory effects of BM-MSCs-derived exosomes on histopathological examination

**Fig. 5:**
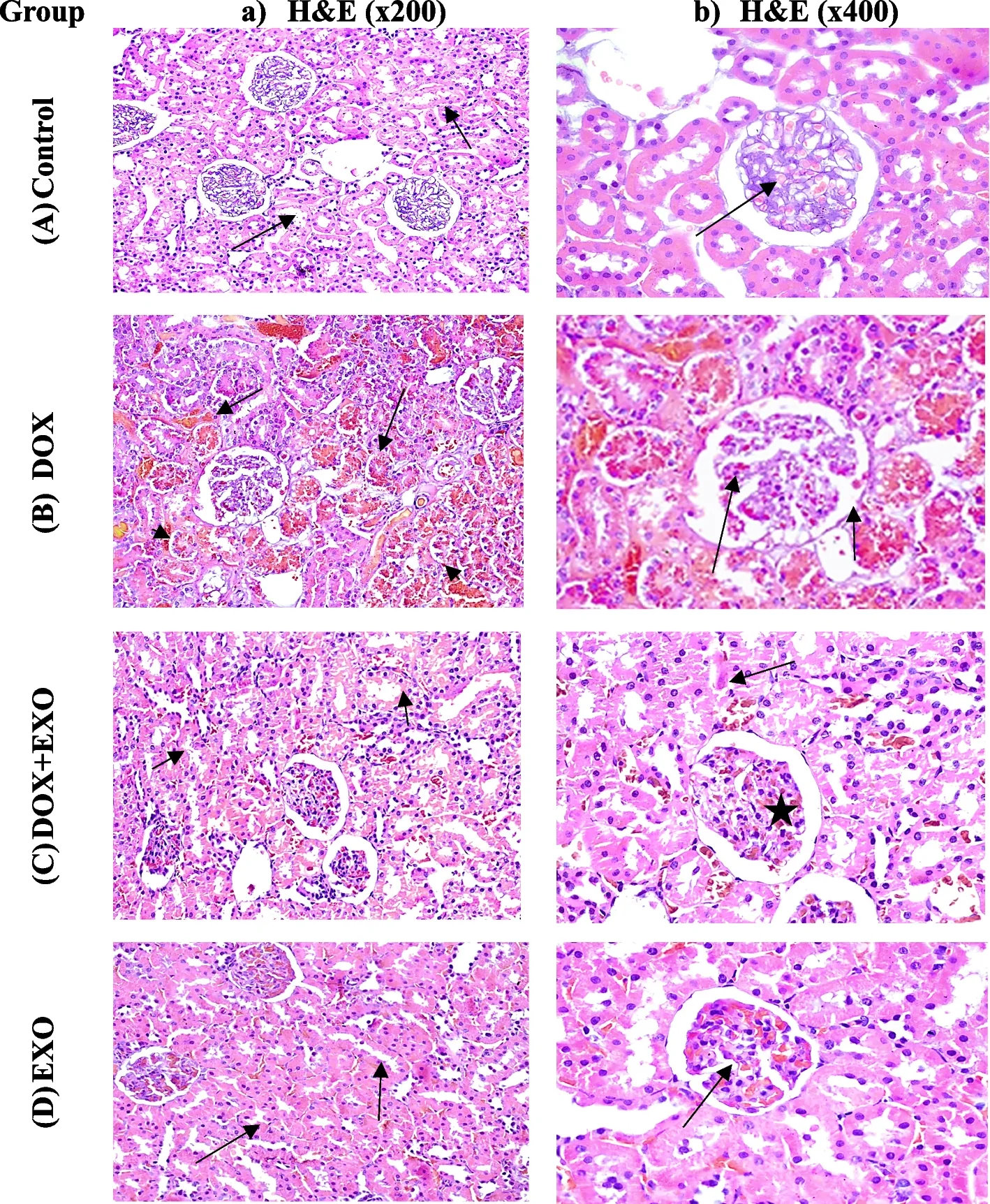
The Modulatory Effects of BM-MSCs-Derived Exosomes on Histopathological Examination. **A** Control group and **D** EXO group: Photomicrograph of kidney tissue section showing (**a**) normal intact proximal tubular epithelial lining (arrow) (× 200), (**b**) circumscribes glomeruli with normal structure of capillary tufts and Bowman's capsule (arrow) (× 400). **B** DOX group: (**a**) shows degeneration of tubular epithelial cells accompanied with narrowing and occlusion of tubular lumen (arrow) and congestion of peri-tubular capillaries (arrow head) (× 200) (**b**) retraction of the capillary tufts and an expansion of Bowman's space (arrow) (× 400). **C** DOX + EXO group: (**a**) swelling of tubular epithelial lining (arrow) (× 200) (**b**) mild congestion of glomerular tufts (star) and interstitial oedema (arrow) (× 400). BM-MSCs: Bone marrow-derived mesenchymal stem cells; EXO: Exosomes; DOX: Doxorubicin; H&E: Hematoxylin & eosin

The ameliorative effects of BM-MSC-derived exosomal treatment on DOX-induced AKI was evaluated by histopathological assessment. The rats in the Control group (Group I) (Fig. 5A) and EXO group (Group IV) (Fig. 5D) showed a typical histological structure with well-defined glomeruli, normal capillary tufts, and intact Bowman's capsule. The renal tubules, both proximal and distal, retained an intact epithelial lining and were regularly arranged, without signs of kidney damage. In contrast, the DOX group (Group II) exhibited various levels of damage to the tubular epithelial cells. Many renal tubules exhibited signs of necrosis within their epithelial linings, along with narrowing and obstruction of the tubular lumen with cellular casts. There was noticeable retraction of the capillary tufts and expansion of Bowman's space, with some glomeruli displaying hypercellularity of their capillary tufts. Additionally, interstitial congestion and hemorrhages were evident, with infiltration by a few inflammatory cells, predominantly lymphocytes and macrophages (Fig. 5B). On the other hand, the DOX + EXO group (Group III) showed significant protection compared to DOX alone. There was only mild swelling of the tubular epithelial lining, along with a few intratubular eosinophilic albumin casts, but no significant necrosis or apoptosis was observed. The glomeruli were well-defined, maintaining a normal arrangement of capillary tufts, and only mild interstitial edema was noted (Fig. 5C). The histopathological scoring of kidney damage was evaluated across different animal groups as summarized in Tables 2 and 3.

**Table 2:** The histopathological score of each rat in each group (*n* = 5)

Rat/No	Control	DOX	DOX + EXO	EXO
1	0	3	0	0
2	0	3	1	1
3	1	3	0	0
4	0	2	2	0
5	0	3	1	0

**Score (0)**: no damage, **(1)**: focal, slight changes (less than 25%), **(2)**: multifocal, significant changes (25%–50%), **(3)**: common, widespread changes (more than 50%)

**Table 3:** Morphological change scores in kidney tissues of each group (*n* = 5)

Morphological change	Control	Dox	DOX + EXO	EXO
Dilatation of Bowman's space	1	3	1	1
Vacuolization in tubule epithelial cells	0	3	2	1
Degeneration in tubule epithelial cells	0	3	2	0
Intratubular hyaline accumulation	1	4	1	0
Vaso-congestion	0	4	0	1

**Score (0)**: no damage, **(1)**: minimal (< % 25), **(2)**: slight (% 25—% 50), **(3)**: medium (% 50-% 75), **(4)**: severe (> % 75)

### The modulatory effects of BM-MSCs-derived exosomes on kidney functions

**Fig. 6:**
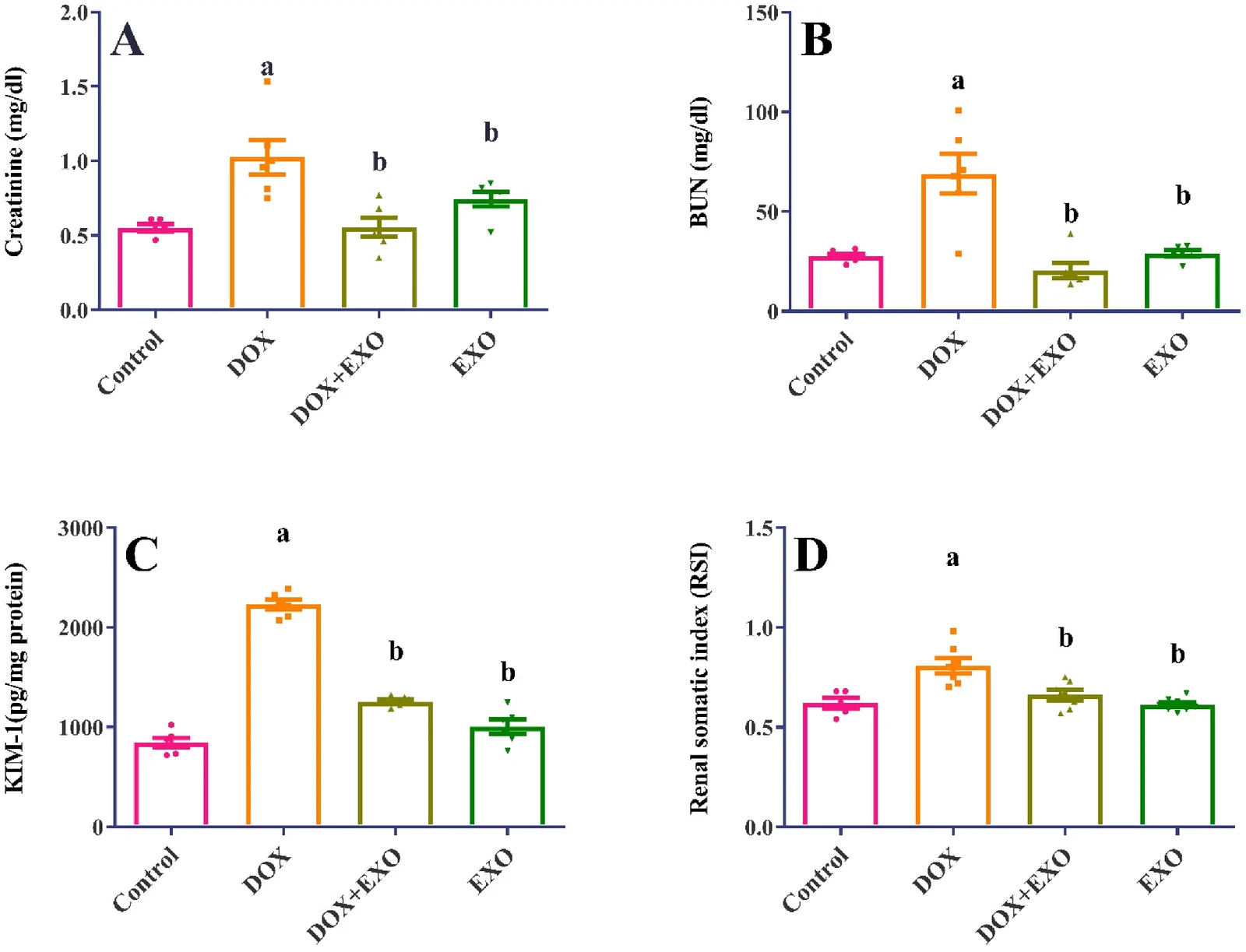
The Modulatory Effects of BM-MSCs-Derived Exosomes on Kidney Function and Kidney Injury. **(A)** Serum creatinine, **(B)** Serum blood urea nitrogen (BUN), **(C)** Kidney injury molecule-1 (KIM-1), and **(D)** Renal somatic index (RSI). DOX Group: Doxorubicin (15 mg/kg); DOX + EXO Group: Doxorubicin followed by a dose of exosomes (100 μg exosomal proteins per rat) the next day; EXO Group: Exosomes only. Data are expressed as mean ± SEM (*n* = 6) using one-way ANOVA followed by Tukey’s post hoc test (*p* < 0.05). "a" indicates a significant difference *vs.* Control group; "b" indicates a significant difference *vs.* DOX group

Kidney function assessment results of Scr and BUN are shown in Fig. 6A and Fig. 6B, respectively. Scr and BUN levels in the DOX-intoxicated group were significantly higher by 86.18% (*p* < 0.0001) and 149.23% (*p* < 0.001), respectively, in comparison to the control group (Group I). Exosomal treatment significantly reduced Scr and BUN levels by 45.92% and 70.32% (*p* < 0.0001), respectively, compared to the DOX-intoxicated group (Group II). The group treated with EXO only (Group IV) showed no significant differences in Scr and BUN compared to the control group (Group I).

### The modulatory effects of BM-MSCs-derived exosomes on kidney injury

The level of KIM-1 was significantly elevated in the DOX-intoxicated group (Group II) by 165.18% (*p* < 0.0001) compared to the control group (Group I). After exosomal treatment, its level significantly decreased by 42.92% compared to the DOX-intoxicated group (Group II) (*p* < 0.001) (Fig. 6C). The RSI was significantly increased in the DOX-intoxicated group (Group II) by 30.41% (*p* < 0.001) in comparison to the control group (Group I). Post-exosomal treatment renormalized the levels of RSI again by 18.2% compared to the DOX-intoxicated group (Group II) (*p* < 0.01) (Fig. 6D). The group treated with EXO only (Group IV) showed no significant differences in kidney injury markers, including KIM-1 and RSI, compared to the control group (Group I).

### The modulatory effects of BM-MSCs-derived exosomes on oxidative stress markers

**Fig. 7:**
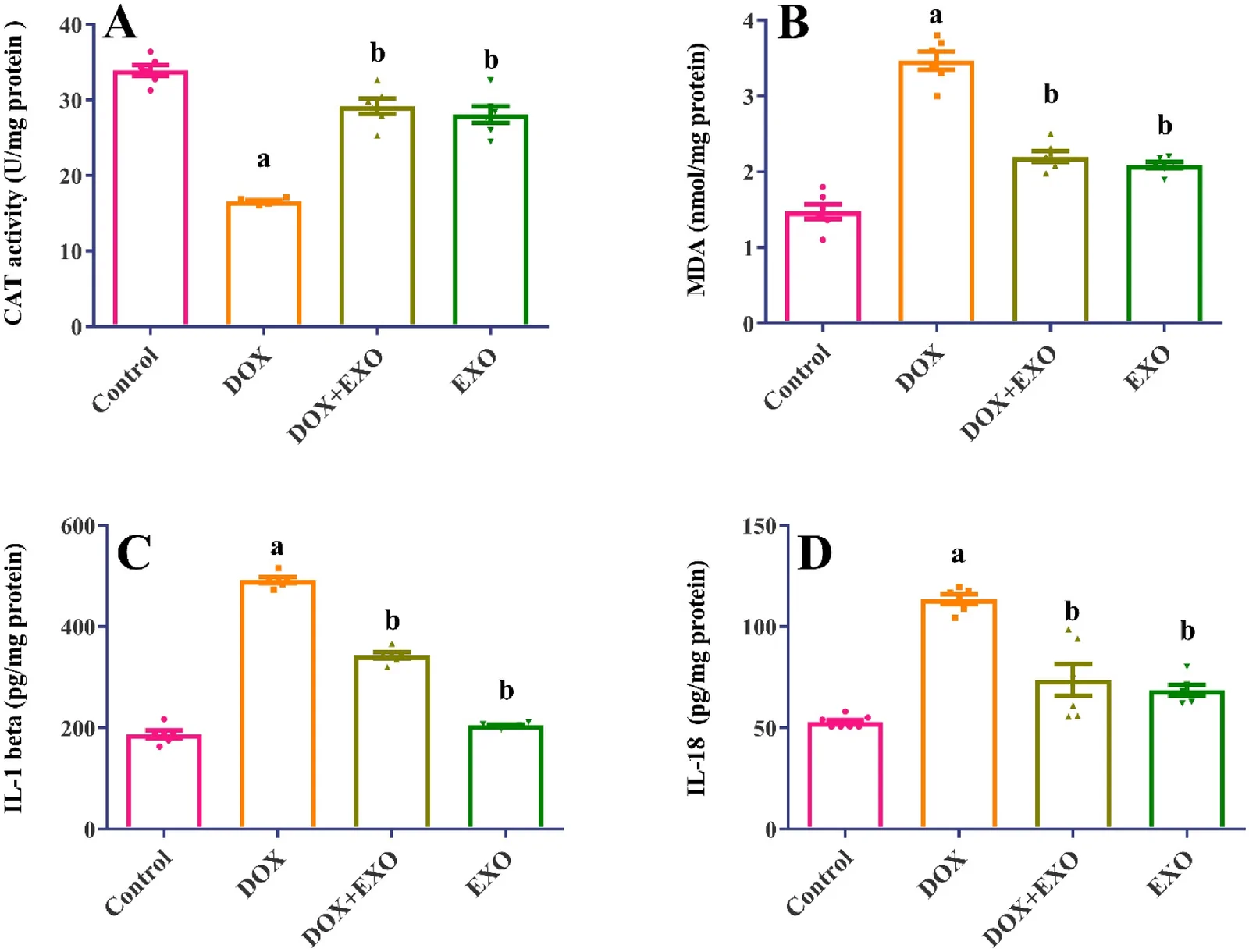
The Modulatory Effects of BM-MSCs-Derived Exosomes on Renal Oxidative Stress and Inflammation. **(A)** Renal catalase (CAT) activity, **(B)** Renal Malondialdehyde (MDA), **(C)** Renal interleukin-1β (IL-1β), and **(D)** Renal interleukin-18 (IL-18). DOX Group: Doxorubicin (15 mg/kg); DOX + EXO Group: Doxorubicin followed by a dose of exosomes (100 μg exosomal proteins per rat) the next day; EXO Group: Exosomes only. Data are expressed as mean ± SEM (*n* = 6) using one-way ANOVA followed by Tukey’s post hoc test (*p* < 0.05). "a" indicates a significant difference *vs.* Control group; "b" indicates a significant difference *vs.* DOX group

The CAT activity was significantly reduced in the DOX-intoxicated group (Group II) by 51.28% (*p* < 0.001) compared to the control group (Group I). Exosomal treatment significantly increased its level by 75.61% (*p* < 0.01) (Fig. 7A). The level of the lipid peroxidation marker MDA was elevated in the DOX-intoxicated group (Group II) by 141.94% (*p* < 0.0001) compared to the control group (Group I). Treatment with BM-MSCs exosomes significantly decreased MDA levels by 35.77% (*p* < 0.001) (Fig. 7B). Remarkably, the control group (Group I) and the EXO only group (Group IV) displayed nearly the same CAT and MDA levels.

### The modulatory effects of BM-MSCs-derived exosomes on inflammatory markers

Both IL-1β and IL-18 were used to measure the inflammatory status of the model. The DOX-intoxicated group (Group II) showed a significant increment in IL-1β and IL-18 levels by 162.65% and 116.81% (*p* < 0.0001), respectively, compared to the control group (Group I). Exosomal treatment was associated with a significant reduction in both IL-1β and IL-18 levels by 30.25% and 35.18% (*p* < 0.0001), respectively, when compared to the DOX intoxicated group (Group II) (Fig. 7C & 7D). The EXO-only treated group (Group IV) showed no significant differences in inflammatory markers, including IL-1β and IL-18, compared with the control group (Group I).

### The modulatory effects of BM-MSCs-derived exosomes on pyroptosis markers

**Fig. 8:**
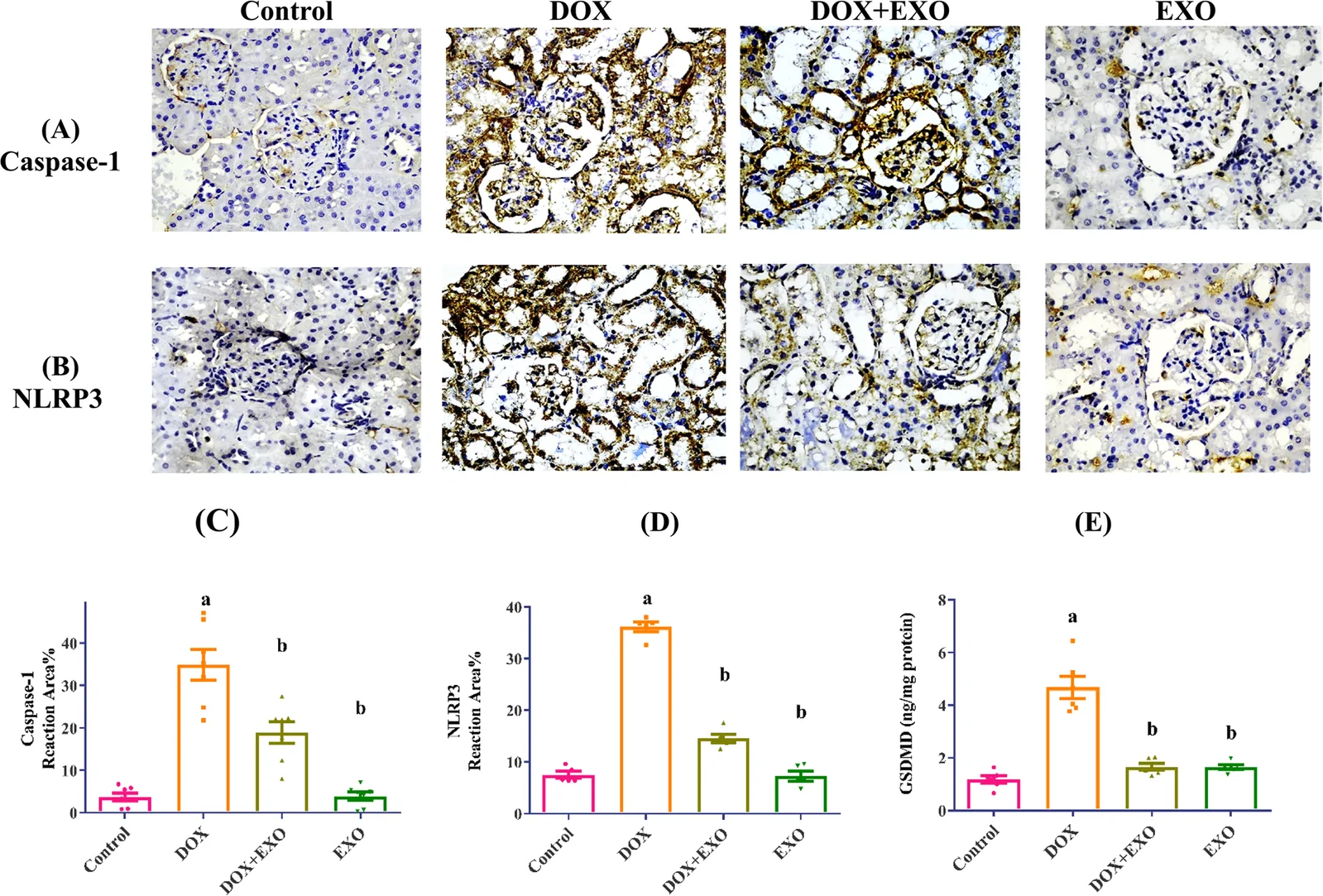
The Modulatory Effects of BM-MSCs-Derived Exosomes on renal pyroptosis. **A** Immunohistochemical staining of caspase-1. **B **Immunohistochemical staining of NLRP3. **C** Renal caspase-1 levels. **D** Renal NLRP3 levels. **E** Renal Gasdermin D (GSDMD) levels. DOX Group: Doxorubicin (15 mg/kg); DOX + EXO Group: Doxorubicin followed by a dose of exosomes (100 μg exosomal proteins per rat) the next day; EXO Group: Exosomes only. Data are expressed as mean ± SEM (*n* = 6) using one-way ANOVA followed by Tukey’s post hoc test (*p* < 0.05). "a" indicates a significant difference *vs.* Control group; "b" indicates a significant difference *vs.* DOX group

As shown in Fig. 8A & B, IHC analysis of renal tissue showed that the DOX-intoxicated group (Group II) had a higher reaction area % for both caspase-1 (Fig. 8C) and NLRP3 (Fig. 8D) than the control group (Group I). On the other hand, exosomal treatment significantly decreased caspase-1 and NLRP3 levels compared to the DOX-intoxicated group. GSDMD levels were significantly increased in the DOX-intoxicated group (Group II) by 295.77% (*p* < 0.01) compared to the control group (Group I). Post-exosomal levels of GSDMD decreased by 64.45% (*p* < 0.01) as compared to the DOX-intoxicated group (Group II) (Fig. 8E). The pyroptosis-associated markers (NLRP3, caspase-1, and GSDMD) showed almost the same levels in the EXO-only treated group (Group IV) compared to the control group (Group I).

### The modulatory effects of BM-MSCs-derived exosomes on miR-146a-5p expression

**Fig. 9:**
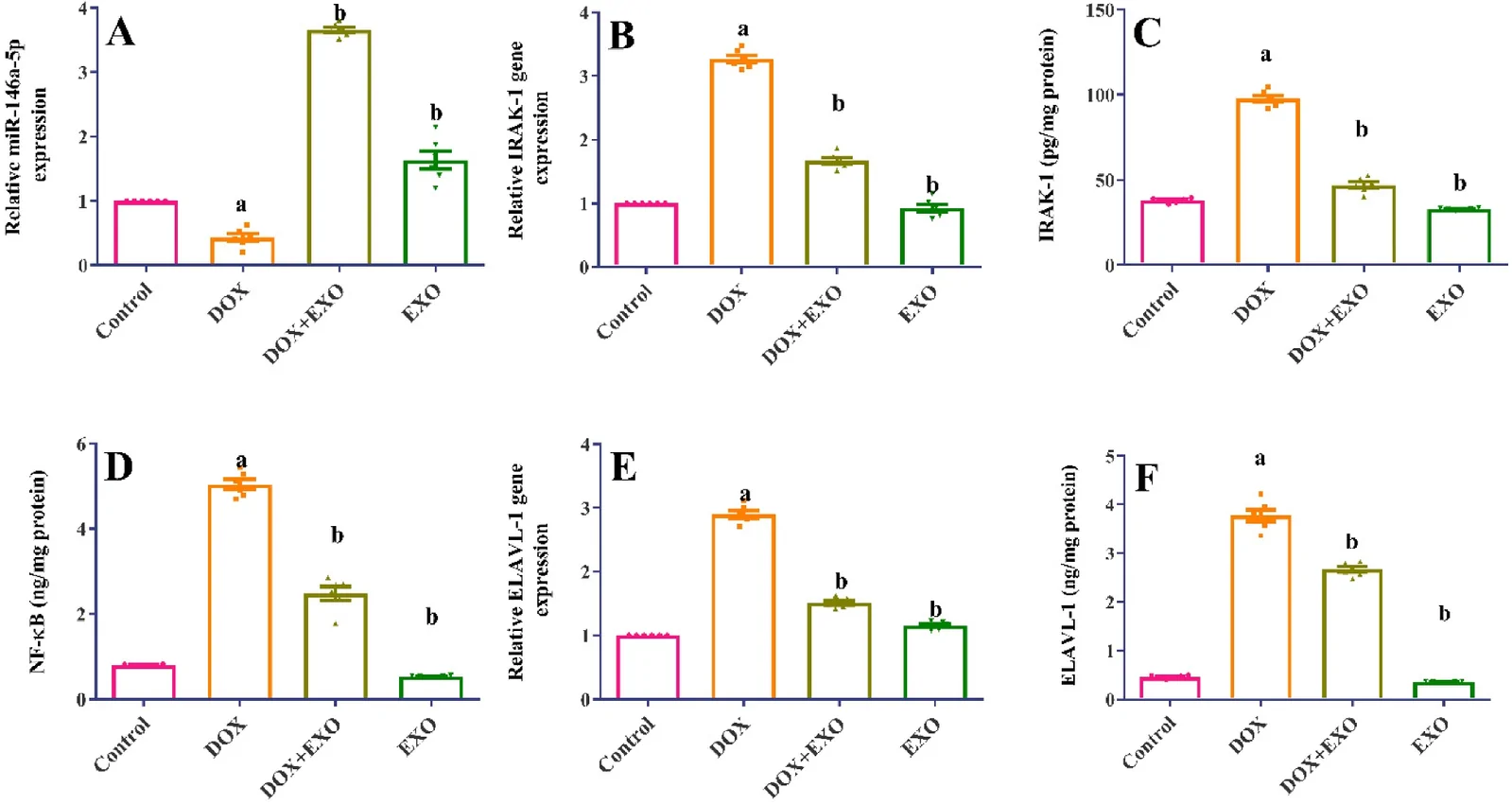
The Modulatory effects of BM-MSCs-derived exosomes on IRAK1, NF-κB, and ELAVL1 (**A**) Relative renal expression level of miR-146a-5p. **B** Relative renal expression levels of IRAK1, **C** Renal IRAK1, **D** Renal NF-κB, **E** Relative renal expression levels of ELAVL1, **F** Renal ELAVL1. DOX Group: Doxorubicin (15 mg/kg); DOX + EXO Group: Doxorubicin followed by a dose of exosomes (100 μg exosomal proteins per rat) the next day; EXO Group: Exosomes only. Data are expressed as mean ± SEM (*n* = 6) using one-way ANOVA followed by Tukey’s post hoc test (*p* < 0.05). "a" indicates a significant difference vs. Control group; "b" indicates a significant difference vs. DOX group

To further investigate the molecular mechanism underlying the ameliorative effect of exosomes on DOX-induced AKI, the fold change of miR-146a-5p expression was measured in all groups. The relative expression of miR-146a-5p was downregulated in the DOX-intoxicated group (Group II) by 56.24% (*p* < 0.05) compared to the control group (Group I). However, treatment with BM-MSCs-derived exosomes resulted in significant upregulation in its relative expression by 728.77% (*p* < 0.0001) compared to the DOX-intoxicated group (Group II) (Fig. 9A). The fold change in miR-146a-5p expression was almost the same in both EXO-only-treated and the control group (Group I).

### The modulatory effects of BM-MSCs-derived exosomes on IRAK1, NF-κB, and ELAVL1

The relative mRNA expression of IRAK1 (Fig. 9B) and its protein level (Fig. 9C) were significantly upregulated in the DOX-intoxicated group (Group II) by 223.33% (*p* < 0.0001) and 157.27% (*p* < 0.0001), respectively, in comparison to the Control group (Group I). However, their levels were reduced by 48.39% and 51.8% (p < 0.0001), respectively, after treatment with BM-MSCs-derived exosomes, compared with the DOX-intoxicated group. Besides, the NF-κB level (Fig. 9D) was elevated in the DOX-intoxicated group (Group II) by 531.56% (p < 0.0001), compared to the Control group (Group I). After exosomal treatment, its level decreased by 50.84% (p < 0.0001). The relative mRNA expression of ELAVL1 (Fig. 9E) and its protein level (Fig. 9F) were significantly elevated in the DOX-intoxicated group (Group II) by 186.67% (*p* < 0.0001) and 728.79% (*p* < 0.0001), compared to the Control group (Group I). Treatment with BM-MSCs exosomes decreased their levels by 48.39% and 29.38%, respectively. The group treated with EXO only (Group IV) showed no significant differences in IRAK1, NF-κB, or ELAVL1 compared to the control group (Group I).

## Discussion

Cancer is the second leading cause of mortality worldwide (Zaorsky et al. 2017). Although DOX demonstrates potent anticancer efficacy against numerous cancer types, its use remains substantially limited by its adverse events, with nephrotoxicity constituting a critical constraint (Wu et al. 2021; Sangweni et al. 2022). Recently, exosomal therapy has emerged as an increasingly promising therapeutic candidate owing to its prominent regenerative and immunomodulatory properties (Wang et al. 2023). In the current study, we investigated the modulatory effects of BM-MSCs-derived exosomes on DOX-induced kidney injury, highlighting the novel crosstalk between miR-146a-5p, oxidative stress, inflammation, and the pyroptosis pathway in an experimental rat model of AKI.

Results of the current study revealed that DOX toxicity caused a deterioration in histopathologic features, with elevations in serum creatinine, BUN, and RSI levels. These results agreed with multiple previous studies (Abd-Ellatif et al. 2022; Rafiee et al. 2020; Soliman et al. 2024). In addition, renal KIM-1 levels were significantly elevated in DOX-intoxicated group, in line with previous studies demonstrating its role as a marker for early detection of kidney injury (El-Ashmawy et al. 2022; Fouad and Ahmed 2021). On the other hand, exosomal treatment in the current study could restore kidney function after DOX-induced toxicity, as evidenced by reductions in Scr, BUN, and KIM-1 levels, in accordance with previous studies (Jin et al. 2023; Xie et al. 2022; Abdelrahman et al. 2020).

Oxidative stress is one of the most well-known mechanisms in the pathogenesis of DOX-induced nephrotoxicity (Amarasiri et al. 2023). Specifically, several oxidoreductases cause one-electron reduction of DOX, resulting in DOX-semiquinone radical formation (Benzer et al. 2018). As a result, DOX enters a cascade of reactions, producing multiple forms of ROS like superoxide anion radicals, hydroxyl radicals, and H₂O₂. These molecules cause oxidative stress, lipid peroxidation, mitochondrial damage, and promote oxidative kidney damage (Abd-Ellatif et al. 2022; Benzer et al. 2018). Similarly, our results showed the relation between DOX-induced kidney injury and oxidative stress, where the DOX-intoxicated group exhibited higher MDA levels, indicating increased lipid peroxidation. Moreover, antioxidant CAT activity was significantly decreased compared to the control group. The exosomal treatment alleviated oxidative stress, as evidenced by decreased MDA levels and increased CAT activity.

Inflammation is considered another key modulator in the pathogenesis of DOX-induced nephrotoxicity (Wu et al. 2021; Zhang et al. 2022). According to our results, both IL-1β and IL-18 levels were elevated, indicating a high level of inflammation, in alignment with previous studies (Al Suleimani et al. 2023; Abd-Ellatif et al. 2022). A possible explanation for that is that ROS stimulate the release of proinflammatory cytokines (IL-1β, IL-18, and TNF-α), with subsequent induction of the inflammatory pathways (Arunachalam et al. 2022; Yerramothu et al. 2018). DOX is responsible for the overproduction of ROS, which activates NLRP3. Subsequently, caspase-1, which was already activated from the inflammasome activation, converts proinflammatory cytokines (pro-IL-1β and pro-IL-18) to their active form (Wu et al. 2021; Li et al. 2019). In the current study, exosomal treatment was associated with a reduction in IL-1β and IL-18 levels, highlighting its anti-inflammatory effect, as shown in previous studies (Wang et al. 2023; Zhang et al. 2022).

Furthermore, our results showed upregulation in pyroptosis-associated markers, including NLRP3, caspase-1, and GSDMD, in the DOX intoxicated group, suggesting that inflammasome-related signaling pathway may be implicated in DOX-induced AKI, in accordance with a previous study (Abd-Ellatif et al. 2022). The pyroptosis-associated pathway is involoved in the pathogenesis of AKI in several studies (Xie et al. 2023; Bai et al. 2022). Xu et al*.* highlighted that NLRP3 activation and inflammation induce sepsis-induced nephrotoxicity in both in vivo and in vitro (Xu et al. 2024). Another study demonstrated that pyroptosis is linked to contrast-induced AKI (CI-AKI), and further showed that Klotho, an anti-inflammatory protein with antioxidant activity, exerts a protective effect against CI-AKI by modulating the NF-κB/NLRP3 pathway (Fu et al. 2023). Pyroptosis is mainly initiated by inflammasome activation and is characterized by cell swelling, membrane pore formation, and cell membrane blebbing, leading to cell lysis and the release of intracellular components (Shi et al. 2022). Caspase-1-dependent canonical pyroptotic death involves four major steps: inflammasome assembly, the activation of pro-caspase-1, the cleavage of GSDMD, and the release of IL-1β and IL-18. After the inflammasome complex is formed, which consists of NLRP3 inflammasome and procaspase-1, the activation of caspase-1 occurs to cause the subsequent activation of GSDMD, the executioner of pyroptosis, which generates pores and induces pyroptosis by mediating the secretion of mature IL-1β and IL-18 (Wang et al. 2022; Eskander et al. 2025).

In addition, DOX intoxication in the current study was associated with higher levels of ELAVL1, an RBP implicated in the regulation of inflammatory and pyroptosis-associated signaling (Li et al. 2017; Jeyabal et al. 2016). A previous study showed that targeting ELAVL1 attenuated the pyroptosis-associated pathway, which exerted a protective effect on diabetic kidney disease in vitro (Lv et al. 2023). These data were consistent with our in silico results, which suggested that ELAVL1 may exert a post-transcriptional regulatory effect on multiple pyroptosis-associated transcripts, further supporting its proposed dual role as both a downstream target of miR-146a-5p and a potential post-transcriptional regulator of NLRP3 and other pyroptosis-related mediators (IRAK1, NLRP3, NF-κB). Numerous studies have demonstrated an association between elevated level of NF-κB and DOX-induced AKI, suggesting that DOX increases ROS overproduction and may activate the NF-κB signaling pathway (Asaad et al. 2021; Mohmmed Hegab et al. 2025) as proved in our results. A recent study demonstrated that high ROS levels activate NF-κB and upregulate the NLRP3 inflammasome. Attenuating oxidative stress reduced the activation of the NF-κB/NLRP3/IL-18 axis, thus mitigating LPS-induced retinal inflammation, further underscoring the crucial role of this pathway in inflammatory injury (El Tabaa et al. 2025). Similarly, our results showed an increase in NF-κB in DOX intoxicated group compared to the control, supporting the involvement of NF-κB-associated inflammatory signaling in DOX-induced renal injury. The relationship between ELAVL1 and NF-κB signaling has been described at multiple levels in the literature. Network-based analyses have identified ELAVL1/HuR and NF-κB as co-central hub proteins within shared interaction networks, with both proteins showing high connectivity to genes involved in inflammatory and oncogenic signaling (Suleiman et al. 2015). Rothamel et al*.* (2021) demonstrated that ELAVL1 binds directly and stabilizes key positive regulators of TLR signaling, including the adaptor protein TRAF6, and NF-κB, thereby supporting pathogen-triggered innate immune activation (Rothamel et al. 2021). This is consistent with its broader role in modulating inflammatory signaling pathways, including those converging on NF-κB (Pascale and Govoni 2012). Taken together, these findings are consistent with our in silico results, which support a potential role of ELAVL1 in regulating inflammatory and pyroptosis-associated signaling.

Our findings also showed upregulated expression as well as elevated protein levels of IRAK1 in the DOX-intoxicated group, which supports an association between IRAK1 and DOX-induced renal injury. IRAK1 is a member of the IL-1 receptor-associated kinase family and plays an important role in TLR/IL-1 receptor-mediated NF-κB signaling, which can promote the transcription of NLRP3, procaspase-1, pro-IL-18, and pro-IL-1β. This, in turn, may contribute to the activation of pyroptosis and inflammatory pathways, in agreement with previous studies (Tie et al. 2024; Hou et al. 2021). IRAK1 has been implicated in other inflammatory diseases (Jain et al. 2014), where a recent study by Li et al*.* unveiled the relationship between IRAK1 and inflammatory pathways, demonstrating the association between IRAK1/NLRP3 signaling and inflammation and cartilage degeneration in osteoarthritis (Li et al. 2021). Alongside, Han et al. reported that IRAK1/NF-κB pathway inhibition could alleviate inflammation and apoptosis pathways in dry eye disease (Han et al. 2023). These results align with our in silico analysis, which suggested the involvement of IRAK1 in TLR/MyD88-dependent NF-κB activation and its potential connection with NLRP3 inflammasome-related pathway, supporting the proposed miR-146a-5p/ELAVL1/IRAK1/NLRP3 signaling axis.

Exosomes facilitate cell-to-cell communication through their cargo, including proteins and metabolites (e.g., amino acids, lipids, DNA, mRNA, and miRNAs) (El-Derany and AbdelHamid 2021; Wang et al. 2024; Lv et al. 2019). It is well established that intravenously administered exosomes do not localize exclusively to a single target organ; where systemic administration typically results in predominant accumulation in the liver and spleen due to rapid clearance by the mononuclear phagocyte system, lungs, and kidneys (Wiklander et al. 2015). Intravenously administered exosomes travel through the circulation and are recruited to sites of injury through injury-related changes in blood vessels and by specific interactions between exosomal surface proteins and signals released at the damaged site. For instance, exosomes were shown to accumulate at ischemic kidney tissue after intravenous injection, guided by an interaction between exosomal CXCR4 and SDF-1α, a signal upregulated at the site of injury; blocking this interaction prevented both the exosomes from reaching the kidney and their protective effect. This target occurred within 30 min and was limited to the injured kidney rather than the healthy contralateral kidney, showing that the injury itself recruits the exosomes (Viñas et al. 2018). Increased vessel permeability and inflammatory signaling at sites of injury are thought to underlie this same pattern more broadly, explaining why exosomes tend to accumulate more in damaged tissue than in healthy tissue of the same organ (Grange et al. 2014). This supports the idea that, in our study, the administered exosomes likely circulated systemically before being preferentially drawn to the site of DOX-induced kidney injury, rather than accumulating there regardless of injury. Since our outcome measures (Scr, BUN, renal histopathology, and kidney pyroptosis markers) are specific to the kidney, off-target exosome distribution to other organs is unlikely to affect these results.

In the current study, expression of miR-146a-5p was downregulated in DOX-intoxicated group and was significantly increased after exosomal treatment. The in silico results (RNA-LOCATE website) supported the reported presence of miR-146a-5p in BM-MSC-derived exosomes, consistent with a previous study (Huang et al. 2022). The anti-inflammatory and anti-oxidative roles of miR-146a-5p have been elucidated in various types of kidney injuries (Fouad and Ahmed 2021; Wu et al. 2021; Li et al. 2020; Aboismaiel et al. 2024). In alignment, exosomal treatment exerted an ameliorative effect in DOX-induced AKI in vivo model, as demonstrated by histological, biochemical, and immunohistochemical analyses. Recent evidence has reported the attenuative role of miR-146a-5p in various renal diseases through the regulation of targets such as TRAF6 and Tfdp2 (Zhang et al. 2022; Wu et al. 2021; Teng et al. 2022). In line with this, the in silico findings identified several molecular targets of miR-146a-5p across three databases (TargetScan, miRDB, and DIANA tools). The results of the functional distribution analysis and KEGG pathways indicated that these predicted targets are associated with multiple pathways, including oxidative stress- and TLR-related pathways, which is consistent with Li et al., who demonstrated that miR-146a inhibits IRAK1, thereby suppressing the TLR signaling pathway (Li et al. 2015). Besides, a low level of miR-146a-5p was shown to be associated with diabetic complications such as diabetic retinopathy and insulin resistance (Rasoulinejad et al. 2021). Moreover, miR-146a-5p has previously been reported to have antioxidant potential (Li et al. 2017), as its overexpression was associated with attenuation of oxidative stress and inflammation in a rat model of diabetic retinopathy by stimulating Nrf2 and suppressing NF-κB and TNF-α (Rasoulinejad et al. 2021). Another reported target of miR-146a-5p related to oxidative stress is TP53-induced glycolysis and apoptosis regulator (TIGAR), as its downregulation in human hippocampal neurons of Alzheimer's patients activates pyroptosis and oxidative stress (Lei et al. 2021). Also, the regulation of NLRP3 by miR-146a-5p was previously reported in a study demonstrating the protective role of miR-146a-5p in necrotizing enterocolitis through targeting NLRP3 (Chen et al. 2021).

Compared with whole-cell BM-MSC transplantation, BM-MSC-derived exosome therapy offers several potential advantages as a cell-free alternative, given their favorable safety profile, storage stability, and simpler manufacturing at scale. Exosomes carry the paracrine signaling molecules responsible for MSCs' major therapeutic effects, without the risks associated with live-cell administration, such as uncontrolled differentiation, tumorigenic potential, immune rejection, and pulmonary microembolism following intravenous infusion (Rani et al. 2015; Nassar et al. 2016). Exosomes are also smaller, more stable during storage, easier to standardize in dose and composition, and less complicated in manufacture and quality control than living cell products (Zubair et al. 2025). However, exosome-based therapy also has limitations compared to cell-based approaches. As exosomes lack self-renewal and differentiation capacity, their therapeutic effect is transient and dose-dependent, rather than providing the sustained paracrine support characteristic of living MSCs (Ma et al. 2020). Collectively, while BM-MSC-derived exosomes represent a promising cell-free alternative to conventional BM-MSC therapy, standardized isolation and characterization protocols, validated potency assays, and further in vivo studies addressing biodistribution and dose optimization will be needed to fully establish their clinical translational potential for preventing or treating chemotherapy-associated nephrotoxicity (Zubair et al. 2025; Cao et al. 2022).

In summary, the current study demonstrated that exosomal administration significantly improved renal function and reduced histopathological damage. Exosomes mitigated oxidative stress and reduced lipid peroxidation, while also suppressing inflammatory mediators and modulating pyroptosis-associated signaling pathways. These renoprotective effects suggest that exosomes exert their therapeutic action through coordinated modulation of oxidative, inflammatory, and inflammasome-related signaling. Caspase-1 and GSDMD were assessed at the level of total protein expression, which together with tissue IL-1β/IL-18 levels, reflect inflammasome-associated inflammatory signaling rather than pyroptosis execution. Independent cell-death assay and cleavage-specific analyses represent an important direction for future validation. In addition, direct confirmation of miR-146a-5p binding to ELAVL1 and IRAK1 *via* dual-luciferase reporter assays would add molecular precision to the bioinformatically predicted interactions already supported by our RT-qPCR and ELISA data. Similarly, gain- and loss-of-function experiments using miR-146a-5p mimics and inhibitors, alongside complementary studies in renal tubular epithelial cells, would help isolate its direct contribution to the anti-pyroptotic effects observed in vivo. Exosome tracking and biodistribution analyses would further enable precise quantification of renal-targeted delivery, building on the renoprotective outcomes demonstrated in the current study. Finally, evaluation of upstream and downstream mediators such as MyD88, IRAK4/p-IRAK4, and TRAF6 would help clarify the full signaling cascade linking IRAK1 to NF-κB and NLRP3 inflammasome activation. A deeper understanding of the mechanisms underlying pyroptosis will provide deeper insight into the pathogenesis of AKI and pave the way for identifying novel therapeutic targets.

## Conclusion

The present study provides evidence supporting the ameliorative role of BM-MSCs-derived exosomes in attenuating DOX-induced AKI, as reflected in improved renal oxidative stress and inflammatory markers, together with reduced levels of pyroptosis-related markers. These changes coincided with upregulation of miR-146a-5p and altered expression of ELAVL1, IRAK1, and NF-κB, supporting a candidate miR-146a-5p/ELAVL1/IRAK1/NF-κB/NLRP3 regulatory axis. Collectively, these findings support the concept that BM-MSC-derived exosomes are a biologically plausible intervention for DOX-associated AKI in preclinical settings. Future studies are warranted for further mechanistic validation, optimizing exosome sourcing, dosing, and delivery systems, and determining their translational potential as a novel strategy for attenuating chemotherapy-induced renal injury.

## Supplementary Information

Supplementary Material 1

Supplementary Material 2

Supplementary Material 3

Supplementary Material 4

Supplementary Material 5

Supplementary Material 6

## Data Availability

All data generated or analyzed during this study are included in this article and its supplementary files.

## Abbreviations

AKI: Acute kidney injury
ANOVA: Analysis of variance
BUN: Blood urea nitrogen
BM-MSCs: Bone marrow-derived mesenchymal stem cells
CAT: Catalase
CASP-1: Caspase-1
cDNA: Complementary DNA
CI-AKI: Contrast-induced AKI
CKD: Chronic kidney disease
DAMPs: Damage-associated molecular patterns
DOX: Doxorubicin
ELAVL1: ELAV-like RNA-binding protein 1
EVs: Extracellular vesicles
EXO: Exosomes
FBS: Fetal bovine serum
GSDMD: Gasdermin-D
H&E: Hematoxylin & eosin
H₂O₂: Hydrogen peroxide
IRAK1: Interleukin-1 receptor- 1 associated kinase 1
IL-1β: Interleukin-1 beta
IL-18: Interleukin-18
IHC: Immunohistochemistry
KIM-1: Kidney injury molecule-1
L-FABP: Liver-type fatty acid-binding protein
MAPK: Mitogen-activated protein kinase
MDA: Malondialdehyde
miRNA: MicroRNA
MSCs: Mesenchymal stem cells
NBF: Neutral buffered formalin
NF-κB: Nuclear factor-kappa B
NGAL: Neutrophil gelatinase-associated l ipocalin
NLRP3: Nucleotide-binding oligomerization domain-like receptor family pyrin domain containing 3
Nrf2: Nuclear factor erythroid 2–related factor 2
PAMPs: Pathogen-associated molecular patterns
PBS: Phosphate-buffered saline
qRT-PCR: Quantitative real-time polymerase chain reaction
ROS: Reactive oxygen species
RSI: Renal somatic index
Scr: Serum creatinine
TEM: Transmission electron microscope
TLR: Toll-like receptor
TIGAR: Tp53-induced glycolysis and apoptosis regulator
TNF-α: Tumor necrosis factor-alpha
TRAF6: Tumor necrosis factor receptor-associated factor-6
